# Solid State Gas Sensor Research in Germany – a Status Report

**DOI:** 10.3390/s90604323

**Published:** 2009-06-03

**Authors:** Ralf Moos, Kathy Sahner, Maximilian Fleischer, Ulrich Guth, Nicolae Barsan, Udo Weimar

**Affiliations:** 1 Functional Materials Laboratory, University of Bayreuth, 95440 Bayreuth, Germany; 2 Siemens AG, Corporate Technology, CT PS 6, 81730 München, Germany; 3 Kurt-Schwabe Research Institute Meinsberg, 04720 Ziegra-Knobelsdorf, Germany; 4 Institute of Physical Chemistry, University of Tübingen, 72076 Tübingen, Germany

**Keywords:** impedance spectroscopy, mixed potential, SnO_2_, Ga_2_O_3_, Kelvin probe, operando

## Abstract

This status report overviews activities of the German gas sensor research community. It highlights recent progress in the field of potentiometric, amperometric, conductometric, impedimetric, and field effect-based gas sensors. It is shown that besides step-by-step improvements of conventional principles, e.g. by the application of novel materials, novel principles turned out to enable new markets. In the field of mixed potential gas sensors, novel materials allow for selective detection of combustion exhaust components. The same goal can be reached by using zeolites for impedimetric gas sensors. Operando spectroscopy is a powerful tool to learn about the mechanisms in n-type and in p-type conductometric sensors and to design knowledge-based improved sensor devices. Novel deposition methods are applied to gain direct access to the material morphology as well as to obtain dense thick metal oxide films without high temperature steps. Since conductometric and impedimetric sensors have the disadvantage that a current has to pass the gas sensitive film, film morphology, electrode materials, and geometrical issues affect the sensor signal. Therefore, one tries to measure directly the Fermi level position either by measuring the gas-dependent Seebeck coefficient at high temperatures or at room temperature by applying a modified miniaturized Kelvin probe method, where surface adsorption-based work function changes drive the drain-source current of a field effect transistor.

## Introduction

Reliable detection of hazardous, harmful, or toxic gases has become a major issue due to more stringent environmental or safety regulations worldwide. Solid state gas sensors present a high potential for applications where the use of conventional analytical systems such as gas chromatography or optical detection (e.g. by infrared radiation) is prohibitively expensive. The interaction between the analyte in the surrounding gas phase and the sensor material is transduced as a measurable electrical signal that most often is a change in the conductance, capacitance, or potential of the active element. According to the respective measurement type, these sensor devices are commonly classified as “potentiometric”, “amperometric”, “conductometric”, and so on.

During the past decades, a multitude of solid state sensors has been designed and brought to market, in particular in safety, automotive, process control, or household applications [1]. For many years, Taguchi-type metal-oxide sensors have been used for the detection of toxic or explosive gases [2]. Alternative sensor concepts for this purpose are based on electrochemical cells with polymer electrolytes. To ensure comfort of living spaces, capacitive polymer sensors measuring humidity are available [3]. Zirconia-based electrochemical cells monitor residual oxygen in automotive exhaust [4] or metal melts [5]. More recent developments comprise zeolite-based ammonia sensors [6] or mixed-potential CO/HC sensors [7].

Nevertheless, research continues to meet the increasing demand for devices that present higher sensitivity, higher selectivity, and improved long-term stability at reduced cost. Due to Germany's cutting-edge pollution control regulations, the design of more efficient gas sensor devices has become a particularly active research field in this country.

In view of the numerous existing reviews on the field of gas sensing (e.g. [8,9]), the present contribution does not aim at giving a repetitive discussion of the well-established chemical gas sensors. Being a review paper in Sensors' special issue “State-of-the-Art Sensors Technology in Germany”, more recent research topics are discussed that have received increased attention within the German sensor community for the past few years. Wherever applicable, the present review follows the common classification of sensors according to the measurand. However, an unambiguous assignment is not always possible for the novel trends discussed here. It should be annotated here that due to space limitations, the present review has been confined to potentiometric, amperometric, conductometric, and impedimetric principles, excluding optical principles or radio frequency-based principles. For the same reason, resonant sensor principles based on surface acoustic wave or bulk acoustic wave devices (SAW, BAW) or on quartz-crystal oscillators are not considered.

## 1. Potentiometric Sensors

Since the potential is not a function of geometry parameters, potentiometric devices are intrinsically independent from the sample dimensions. Various types of potentiometric sensors have been investigated for decades. *Equilibrium or Nernstian* electrochemical cells based on solid electrolytes, for example, may be employed for sensing chemical species in a very selective and accurate way. Among these candidates feature the stabilized zirconia air-to-fuel (or lambda) sensor for automotive applications [10] or the oxygen sensor (also zirconia-based) used in steelmaking [5]. In addition, several *Non-Nernstian* or mixed potential devices measuring gases in non-equilibrium conditions have been investigated. Very detailed reviews on electrochemical ceramic sensors discussing the respective mechanisms are available for example in [11-13]. The present section first presents recent trends developed for both subgroups. This discussion is followed by the discussion of a novel potentiometric sensor principle based on the direct thermoelectric effect.

### 1.1. Nernst-type Sensors: Basic Considerations

Potentiometric sensors using solid electrolytes are suited to measure gas components in different gas phases and liquid melts (e.g. molten steel):

- Gas mixtures that contain free oxygen beside inert gases, e.g. O_2_, N_2_
- Gas mixtures that are in chemical equilibrium, e.g. water gas
- Dissolved oxygen in molten metals (e.g. steel and copper)

The measurement of combustible volatile components in gases or as dissolved species in different liquid media gains importance, due to their widespread utilization in different energetic, chemical and biochemical processes and also due to the intensification of requirements for safety and quality control of technical, chemical and biochemical processes. These sensors are highly selective for certain components in a broader matrix of other gases, long-term stable and maintenance-free over years. Furthermore, they respond fast (ms-timescale) and are therefore an attractive tool in control loops. As a solid electrolyte gas-tight sintered ceramics in form of tubes, discs, planar substrates or thick films consisting of stabilized zirconia (e.g. yttria stabilized zirconia; YSZ) are utilized. Due to replacing zirconium ions by lower valent yttrium ions, oxide ion vacancies are generated. With increasing temperature, the transport of oxide ions (O^2^) becomes more and more possible. As a result, the electrical (ionic) conductivity increases exponentially with increasing temperature.

**Figure 1.**
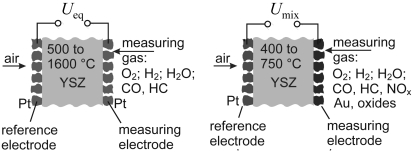
(a) potentiometric Nernst sensor (left). (b) mixed potential sensor (right).

Potentiometric sensors for free oxygen and equilibrium oxygen (see Figure 1a) are very common in gas phases with established thermodynamic equilibria (e.g., *p*(CO_2_)/*p*(CO) or, generally, the ratio of partial pressures of burnt and unburnt components). For that purpose, highly porous Pt is used as an electrode material (Figure 1a). Symbolized by O_2_(φ′_O2_), Pt | (ZrO_2_)_0.84_(Y_2_O_3_)_0.08_ | Pt, O_2_(φ″_O2_), such cells can be regarded as oxygen concentration cells. Depending on their electrode material, they work in a broad range of temperature (400 °C to 1,600 °C) and oxygen partial pressures (10 bar to 10^-20^ bar). As a test probe, such sensors can be applied directly in high temperature processes, so that *in-situ* information may be obtained in real time. The generation of a cell voltage can be explained as follows: Oxygen will move from the side with high partial pressure toward the side with low partial pressure. That is impossible provided that the solid electrolyte is gas tight. In the tendency, oxygen can only move through the electrolyte as an oxide ion (O^2-^). Oxygen takes up four electrons from electronic conducting material (here Pt) and moves through the electrolyte (cathodic process, in right direction). On the side with lower oxygen partial pressure the reverse process takes place (anodic process, in left direction).

The cell reaction is the transfer of oxygen from one side to the other. Using the *Kröger-Vink* notation for defects this can be written: ½ O_2_(g) + V_O_^••^(YSZ) + 2e^-^(Pt) ↔ O_O_ (YSZ). In the case of an electrochemical equilibrium, the measured open circuit voltage or equilibrium voltage *U*_eq_ or *E* or *emf* can be calculated by the Nernst equation:

(1) $$E=-U_{\text{eq}}=\frac{\text{RT}}{4\;F}\text{ln}\frac{p_{o_{2}}^{\prime }}{p_{o_{2}}^{\prime \prime }}$$

Therefore, such sensors are called Nernstian sensors. Air with defined humidity is used as a reference, e.g. air with 50 % r.h. contains 20.63 vol.% O_2_. In reducing gases in the chemical equilibrium (e.g. H_2_/H_2_O; CO/CO_2_; water gas), the oxygen partial pressure is determined by the mass law constant *K*_p_ and this in turn depends on the temperature. In the case of CO/CO_2_-mixtures, the cell voltage is obtained by inserting the temperature function of log *K*_p_ = 4.505 − 14700 K/T into the Nernst equation.

With pure oxygen as *p*″_O2_:

(2) $$\text{E}(\text{CO},{\text{CO}}_{2})/\text{mV}=-0.0496\frac{\text{T}}{\text{K}}\text{lg}{\left[{\text{K}}_{\text{p}}\left(\frac{{\text{p}}_{{\text{co}}_{2}}}{{\text{p}}_{\text{co}}}\right)\right]}^{2}$$

(3) $$\text{E}(\text{CO},{\text{CO}}_{2})/\text{mV}=1458.4-\left[0.447+0.0992\log\left(\frac{{\text{p}}_{{\text{co}}_{2}}}{{\text{p}}_{\text{co}}}\right)\right]\text{T}/\text{K}$$

**Figure 2.**
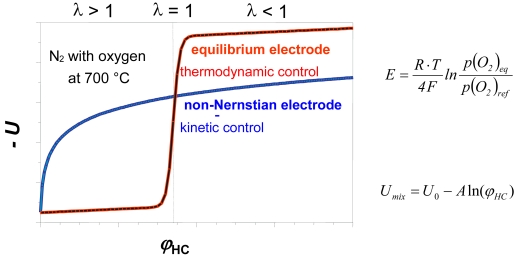
Scheme of the voltage characteristics of an idealized hydrocarbon electrode vs. Pt-air reference electrode in hydrocarbon containing mixtures. *λ* denotes the normalized air-to-fuel ratio (*λ* > 1 denotes a rich and *λ* < 1 a lean exhaust gas). Adapted from [12].

For H_2_/H_2_O and H_2_/H_2_O, CO/CO_2_ (water gas) similar thermodynamically based equations can be derived [14]. The main application field is the fast measurement of oxygen concentrations in liquid metals and gases, such as flue gases of combustion in steam boilers, in glass and ceramic making industries. By combination of sensor signals with stoichiometric and thermodynamic relations, a complete determination of gas phases under equilibrium conditions is possible [15]. Potentiometric solid electrolyte cells possess the first place among all produced chemical sensors, both with respect to number and the resulting economic effects of their application. The lambda probes for the detection of the oxygen/fuel ratio are also oxygen concentration cells (Figure 2). The signal of a lambda probe (red curve) is low in the case of oxygen excess (lean mixtures, left side, *λ* > 1) and high at an excess of fuel (rich mixtures, *λ* < 1, right side). Potentiometric cells can be used to investigate flames *in-situ* in order to determine the border line of the combustion [16]. Oxygen concentration cells can also be constructed with other solid electrolytes (e.g. sodium ion conductors) provided that the ionic transport number is one. But the response time of such sensors is much slower than that of sensors using oxide ion conducting electrolyte.

### 1.2. Nernst-type Sensors: Novel Materials

**Figure 3.**
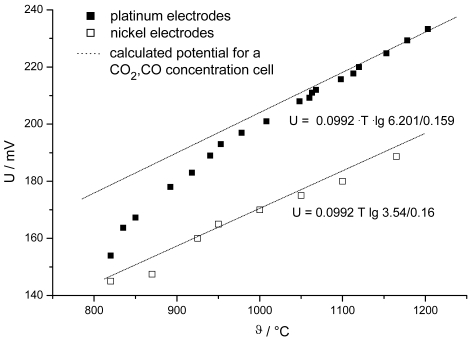
*emf* of the gas cell (I) CO, CO_2_, Pt |YSZ| Pt,CO,CO_2_ (II). Adapted from [12].

Usually, porous Pt is used as an electrode material. A Pt wire in a close contact to the solid electrolyte as a ligature suffices for high temperatures (> 1,100 °C). In the case of CO/CO_2_-gas mixtures, deviations from the Nernst equation can be observed when the measured at less than 1,000 °C (Figure 3). On Ni-electrodes, however, the thermodynamic *emf* is obtained down to 800 °C.

To measure in the equilibrium state at low temperature, very fine Pt, mixed oxides of the perovskite type (e.g. La_0.2_Sr_0.8_MnO_3_) not only as a sintered layer but also as a powder [17], or Ag can be used. Catalytic non active electrode materials (LaCrO_3_) allow to measure oxygen beside hydrocarbons in a non-equilibrium gas mixtures [18-20].

Whilst the application of oxygen sensors in different branches of industry is state of the art [21], Nernstian solid electrolyte sensors for SO_2_ and NO_x_ are in an early stage. The activities respecting potentiometric CO_2_ sensors which are commercially available are summarized more recently [22].

### 1.3. Mixed-potential Sensors: Latest Developments

For a test gas containing hydrocarbons C_m_H_n_ and an excess of oxygen, the cell voltage follows Equation 4 in the case of an electrochemical equilibrium [23]. The resulting graph is shown in Figure 2, red curve.

(4) $$E=\frac{\text{RT}}{4F}\text{ln}\frac{\left[p\left(O_{2}\right)-\left(m+n/4\right)p\left(C_{m}H_{n}\right)\right]}{p{\left(O_{2}\right)}_{\text{ref}(\text{air})}}$$

The measurement of gas components like hydrocarbons (HC) or nitric oxides (NO_x_) in non-equilibrated gas phases has become increasingly important. Depending on the electrode material, the gas components do not equilibrate on the measuring electrode at temperatures < 700 °C. Thus, gas components that are not thermodynamically stable are electrochemically active. In HC and O_2_ containing gases, at least two electrode reactions can take place: the electrochemical reduction of oxygen and the electrochemical oxidation of hydrocarbons [12]:

- Oxygen reduction: 9/2 O_2_(g) + 9V_O_^••^ + 18e^-^ ↔ 9O_O_^x^ (YSZ)
- Hydrocarbon reduction: C_3_H_6_(g) + 9O_O_^x^ (YSZ) → 3CO_2_(g) + 3H_2_O(g) + 9V_O_^••^ + 18e^-^
- Overall reaction: 9/2 O_2_(g) + C_3_H_6_(g) → 3CO_2_(g) + 3H_2_O(g)

The measured open circuit voltage does not obey the Nernst equation. Therefore, such electrode behavior is often referred to non-Nernstian electrodes (or mixed potential sensors), see Figure 1b. A mixed potential sensor combines an oxygen electrode as a reference and a second electrode, which shows a low oxygen sensitivity but a high hydrocarbon sensitivity. Both electrodes may be exposed to the analyte gas. Alternatively, the sensor can operate with an oxygen electrode exposed to air as an external reference. According to the theory by Miura [24] based on the Butler-Volmer equation, the cell voltage mainly depends logarithmically on the concentrations (Figure 2, blue curve):

(5) $$U_{\text{mix}}=U_{0}-\text{A ln}(\varphi_{\text{HC}})$$

The effect of hydrocarbon sensitivity of Pt electrodes at relatively low temperatures was described as early as 1981 [25]. The mixed potential of such solid electrolyte electrodes is contrary to that of electrodes in aqueous solution very stable and reproducible [26]. The voltage response and the response time depend mainly on the electrode material, its catalytic and electro-catalytic properties. In order to search for suitable materials, the voltage response and the catalytic activity have to be investigated. For screening, it is useful to investigate the deviation from the Nernst equation.

**Figure 4.**
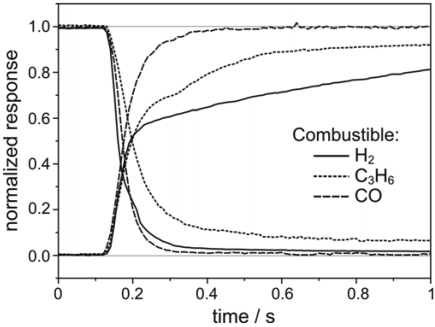
Normalized response of a miniaturized planar gas sensor at 650 °C. Cell symbol: Au,NiO/YSZ/Pt with Au,NiO composite mixed potential electrode (YSZ, yttria stabilized zirconia) Reprinted from [34] with permission from Elsevier.

In the last few years, a variety of materials were investigated with respect to their sensitivity and selectivity as mixed potential electrodes. In most cases, the increasing catalytic conversion of combustibles prevents the appearance of mixed potentials above 800 °C. On the other hand, Wang *et al.* [27] have found a high sensitivity for NO_x_ using Rh-NiO electrodes, even at 800 °C. One of the most intensively discussed and investigated questions concerning non-Nernstian solid electrolyte sensors is that of the electrode process with most relevance for the signal [28,29]. Besides potentiometric measurements, a variety of other investigation methods like amperometric, impedimetric [30-32], catalytic [26] and response measurements were utilized to answer this key question. It seems that mixed potential theory as well as chemisorption processes, connected with changes in the Fermi level of the electrode material [33] can contribute to the sensing mechanism. The response time (450 °C … 750 °C) depends on the kind of gas [34] and is comparable with that of an O_2_, Pt/YSZ-electrodes as shown in Figure 4. The shape of different response curves suggests that different time steps depending on gas composition are involved at the same electrode.

Up to now, many questions concerning the complex behavior of electrode materials in different gases are still open. Therefore, the trial-and-error search for new electrode materials is still state of the art for this type of gas sensors. A lot of different electrode materials are under investigation especially in Miura's group [35-37]. Nevertheless, investigations of the Guth group show [26] that the sensitivity of different Au oxide composite electrode materials (e.g. Au, Nb_2_O_5_) for one combustible may be correlated with the catalytic activity of these electrode materials for oxidizing this combustible. In the case of mixed oxide electrodes (La_0.6_Ca_0.4_Mn_1-x_Me_x_O_3_ Me = Co, Ni), it seems to be a correlation between the voltage response and the kind of conductivity (p- or n-type) [32]. Meanwhile, mixed potential sensors are commercially available to detect the breakthrough of hydrocarbons in air cleaning filters [38] or to monitor the metabolism in biochemistry [39].

### 1.4. Direct Thermoelectric Sensors: a Novel Potentiometric Principle

A novel potentiometric principle has been investigated recently for both equilibrium oxygen sensing and detection of combustibles: the direct thermoelectric principle [40,41]. In resistive gas sensors that are based on semiconducting oxide films, the film resistivity changes due to redox reactions of the analyte with the sensor material (confer Section 3). These resistivity changes are either governed by surface effects at temperatures below 500 °C, like in n-type semiconducting SnO_2_ [2] or by volume effects occurring mostly above 700 °C. In all cases, the electronic charge carrier density (electrons, holes) changes when the material is exposed to the analyte. A major disadvantage of these resistive devices is the dependency of the measurand “sensor resistance” on the film geometry and morphology. For that reason, the measurand is also affected by cracks in the film, by abrasion of the film, or by sintering of the film during operation. Whereas the geometry dependency is crucial for reproducible manufacturing, the latter issues strongly affect long-term stability. In a a direct thermoelectric sensor device, these problems are overcome [40,41]. Here, the Seebeck coefficient *ε* of the material is directly measured. In principle, *ε* describes the position of the Fermi-level in the band scheme and hence, it is correlated with the concentration of the electronic charge carriers in semiconducting materials. For example, in n-type semiconducting oxides, *ε* is related with the electron density *n* by Equation 6 [42]:

(6) $$ɛ=-\frac{k}{e}\cdot \left(\text{ln}\frac{N_{C}}{n}+A_{\text{e}}\right)$$

In Equation 6, *k* is the Boltzmann constant, *e* the electron charge, *N*_C_ the effective density of states in the conduction band, and *A*_e_ is a scattering mechanism-dependent transport coefficient for electrons. Unlike the conductivity, *ε* is independent from the geometry of the gas sensitive material. Despite the relationship between Seebeck coefficient and the conductivity of metal oxides had been evaluated very early by Jonker [43], only scattered works are known implementing this principle as a sensor device, e.g. [44-46].

A basic sensor setup comprises a heater to bring the whole device to operation temperature, insulating layers, and a heater providing a modulated temperature gradient Δ*T* over the gas sensitive film. The measured thermovoltage of the material, *V*_S_, is a function of the Seebeck coefficient of the film material *ε* and of the applied temperature difference Δ*T*:

(7) $$V_{\text{S}}=({ɛ}_{\text{lead}}-ɛ)\Delta T$$

*V*_S_ includes the usually known or at least constant thermovoltage of the leads *V*_lead_ = *ε*_lead_Δ*T* Knowing *V*_S_ and Δ*T*, the Seebeck coefficient of the sample *ε* can be derived. Typically, Δ*T* is modulated periodically and *V*_S_(Δ*T*) is measured. *ε* is deduced from the slope of all data points in the *V*_S_ vs Δ*T*-plot by applying Equation 7.

On the one hand, this principle is suitable for oxygen sensors. Here, the electron and hole densities in the equilibrium are determined by the oxygen partial pressure, *pO_2_*, of the surrounding atmosphere. As expected from defect chemistry in the pure n-type and p-type regime, only a slight dependency of *ε* on *pO_2_* can be found [47,48]. Suitable materials are almost all semiconducting oxides, if their oxygen diffusion coefficient is high enough for a fast sensor response, and if a *pO_2_*-variation affects significantly the mobile electronic charge concentrations. For instance, electrode materials for SOFC might not be suitable for that purpose, since their charge carrier density does not change markedly with *pO_2_*. Examples for realized sensor devices to measure in the pure p-type regime are shown in [40] using SrTi_1-_*_x_*Fe*_x_*O_3-δ_, an excellent material for resistive oxygen sensing [49] or hydrocarbon sensing at lower temperatures [50]. However, slopes of only 52 μVK^-1^ per decade *pO_2_* could have been achieved. Much more sensitive devices can be obtained, if one shifts the S-shaped *ε*(*pO_2_*)-curve by appropriate doping into the intrinsic regime, where donors and acceptors compensate mainly itself in the *pO_2_*-range of interest. Then, a slight increase in the *pO_2_* is sufficient to shift *ε* from negative values to positive ones. Rettig succeeded in preparing sensors with an intrinsic semiconductor showing almost linear slope in the *ε*(*pO_2_*)-plot of 186 μVK^-1^ per decade *pO_2_* [51].

**Figure 5.**
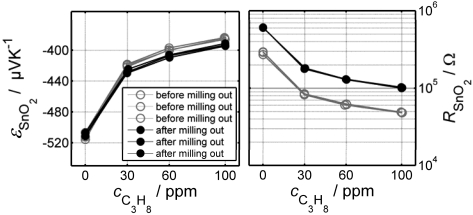
Seebeck coefficient (a) and resistance (b) of a SnO_2_ gas sensor before and after milling out a big part of the gas sensitive film (from [52]). With permission from IEEE (© 2007 IEEE).

On the other hand, the direct thermoelectric principle has been successfully tested for the detection of reducing gases like hydrocarbons using doped SnO_2_ [45,52]. The huge advantage of the direct thermoelectric gas sensors is impressively demonstrated in Figure 5 [52]. Both the Seebeck-coefficient and the resistance of a gas sensitive SnO_2_-film were measured under different propane concentrations. A part of the film was deliberately milled out. The sensor was measured again under the same conditions. Obviously, the resistance of the gas sensitive film increased by about 100 % after milling out a part of the gas sensitive film (sensor reading 28 ppm instead of the true value of 100 ppm). The Seebeck coefficient, however, decreased only by about 5 %; i.e. the sensor reading is 80 ppm propane instead of the true value of 100 ppm.

The effect of the morphology and the influence of dopants was evaluated numerically [51]. Utilizing fine-grained intrinsic or slightly donor-doped materials would be a good choice, since in that case the sensitivity of the thermoelectric gas sensor reaches a high value and the sensor resistance is almost not affected by the analyte concentration. A direct thermoelectric sensor made of such a material would have the benefit that by measuring both the thermopower and the resistance, the resistance value can serve as a temperature signal or if the temperature is known, it could indicate sensor poisoning.

Not only electronic conductors can be employed as materials for direct thermoelectric gas sensors. It has been shown several years ago that the total thermopower *ε* of an electrochemical cell with Pt electrodes that are separated by an ionic oxygen conductor can be written as [53]:

(8) $$ɛ=(ST)-\frac{k}{4e}\cdot \ln(p_{o_{2}})=\frac{Q_{O^{2-}}^{•}}{2eT}-{ɛ}_{Pt}$$

Herein, *S*(*T*) is the entropy term, *Q*^•^_O2-_ is the heat of transport of the oxygen ions, and *ε*_Pt_ the thermopower of the Pt electrodes. In a first order approximation, these three terms can be regarded as constant and a theoretical sensitivity *s* of the thermoelectric cell can be derived:

(9) $$s=\frac{dɛ}{d\log(p_{o_{2}})}=-\frac{k}{4e}\ln10$$

This amounts to a slope of *s* ≈ -50 μVK^-1^ per decade *pO_2_* and is in the same order of magnitude as for semiconductor materials.

**Figure 6.**
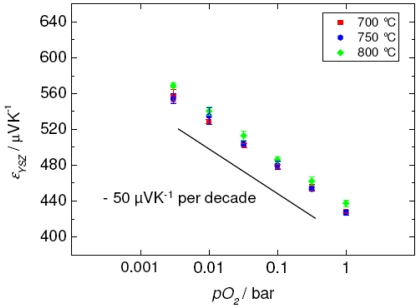
Sensor signal vs oxygen partial pressures for three different temperatures. Please note the very low temperature dependency. Figure adapted from [54]. Reprinted from [54] with permission from Elsevier.

The first implementation of such a sensor device is reported in [54]. 8 mol% Y_2_O_3_ stabilized zirconia, YSZ, was used for the membrane material. From the results in Figure 6 it is obvious that the expected sensitivity is reached. Astonishingly, almost no temperature dependency of *ε* occurs, indicating that the three *pO_2_*-indpendent terms have either a negligible temperature dependency or their temperature dependency compensates each other. Additionally, no cross sensitivities towards NO, H_2_, H_2_O, CO, CO_2_ and HC are observed.

These results deserve a comparison with the conventional potentiometric lambda-probe as described in [10] or [4]. The conventional lambda-probe has the clear advantage of a sensitivity of about 50 mV per decade *pO_2_* when operated at 735 °C. Even if one applies a Δ*T* of 50 °C over the direct thermoelectric gas sensor, a sensitivity of only 2.5 mV per decade *pO_2_* occurs. This disadvantage competes with the simpler sensor setup. No reference atmosphere is required and very tiny samples with a fast light-off time requiring only very low power for operation seem feasible. Nevertheless, it is to emphasize that the direct thermoelectric gas sensor principle is in an early research state far away from market ripeness.

## 2. Amperometric Sensors

In amperometric gas sensing devices, the reaction of an analyte at an electrode generates a current which is then measured commonly at a fixed applied potential. A linear relationship between current and concentration is observed. Reference [55] gives a detailed review on amperometric gas sensor devices, encompassing solid polymer electrolyte (SPE) sensors based on Nafion as well as on sensor cells based on doped zirconia, i.e., two-chamber devices for the simultaneous detection of NO and O_2_ and oxygen pumping cells.

To further reduce cross-interference of other analytes on the sensor signal, the use of highly selective biological components (e.g., enzymes or antigene/antibody pairs) has received increased attention [56]. Due to their specific reaction sites following the “lock and key” model, these biocomponents only interact with particular analytes (also called substrates). Hence, they are ideal candidates for the preparation of selective electrochemical sensing cells. The cell output, an electrical current, can be linked to the analyte concentration.

Due to stability issues of the biological agents, most of the corresponding amperometric devices are applied exclusively in the liquid phase for the detection of solutes. One example is the well-known glucose sensor based on glucose oxidase for monitoring diabetes. More novel biosensor concepts, however, aim at the direct electrochemical detection in the gas phase. Mitsubayashi *et al.* were the first to report gas-phase biosensors for various analytes ranging from ethanol and formaldehyde to trimethylamine, methylmercaptan, and acetaldehyde, e.g. Refs. [57] or [58]. In this case, the sensitive enzyme was immobilized on a Pt-coated PTFE membrane.

In Germany, similar gas-biosensors for formaldehyde, ethanol, and phenol sensing were studied at the University of Bayreuth. For each of the analytes, an appropriate enzyme/gas-diffusion electrode combination was investigated, and sub-ppm level detection was successfully demonstrated [59,60]. The sensor devices consisted of a liquid compartment containing the enzyme and further organic components in a buffered electrolyte system. From the gaseous environment to be monitored, gases diffuse into the liquid reaction volume via a PTFE membrane. In addition, the authors showed that despite the high intrinsic specificity of the enzyme itself, all components of the complex biosensor system need to be taken into account when studying cross-interferences [61]. In the case of the formaldehyde sensor, for example, a low interference of CO_2_ and NO on the sensor signal was observed. This could be linked to either mediator or electrode interactions, and could be reduced by a careful choice of these components.

In a continued study, the initial sensor setup was miniaturized by applying laser micromachining and low-temperature co-fired ceramics (LTCC) technology [62]. The organic diffusion membrane was replaced by a porous metalized ceramic electrode. A reusable hybrid sensor platform was achieved.

## 3. Conductometric Sensors

Metal oxide semiconducting sensors of the resistive or conductometric type have been thoroughly investigated for decades. In the 1950s and 1960s, various research groups reported that some metal oxides change their conductivity significantly when exposed to reducing gases at high temperatures around 400 °C [63-65]. Soon after this discovery, first sensor devices based on tin oxide were commercialized, and the research was expanded to other metal oxide candidates. Numerous reviews on gas sensor devices of the so-called Taguchi type are available [66-69].

The novel trends reviewed in the present section start with n-type and p-type semiconductors. Main focus of the recent research work in this field is to understand the sensing mechanisms in a qualitative and quantitative way with respect to further sensor improvements. Recently, research on zeolites for gas sensor application came up due to the excellent selectivity that can be obtained as a result of the framework structure of these aluminosilicates.

The German key gas sensor labs also work on novel deposition techniques, which may supplement the common thick and thin film methods.

### 3.1. Novel Trends in n-type Semiconducting Sensors

#### 3.1.1. Operando Studies

**Figure 7.**
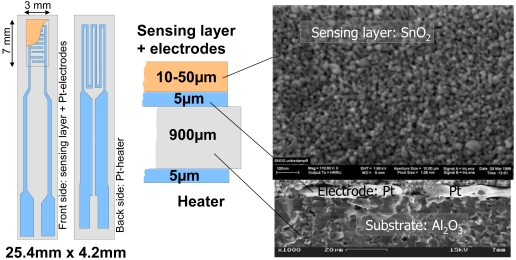
Typical samples used in the operando studies.

Recognizing that one of the major problem encountered by the R&D in the field of gas sensors is the lack of experimental information (phenomenological and spectroscopic) that is gained under the same conditions in which the sensors are operated and on samples that are similar to real sensors, the group at the University of Tübingen established a battery of electrical and spectroscopic techniques able to do exactly that. For the semiconducting metal oxides based ones, an example of the used samples/sensors is presented in Figure 7. The sensing layers are screen-printed or drop coated porous thick films of polycrystalline metal oxides. The substrates are made of alumina provided with thick film Pt electrodes and heaters, which is also how most commercial sensors are constructed. In the experimental setups, sensors are characterized in normal working conditions (normal pressure – 1 bar; dynamic system for dosing of the gaseous composition of the sensor ambient - r.h. between 0 and 90 %, target gases concentration between few ppb and 100 %, total flow between 10 and 1,000 ml/min; adjustment of sensor temperatures between r.t. - 500 °C; “operando” techniques), see **Error! Reference source not found**.7.

**Figure 8.**
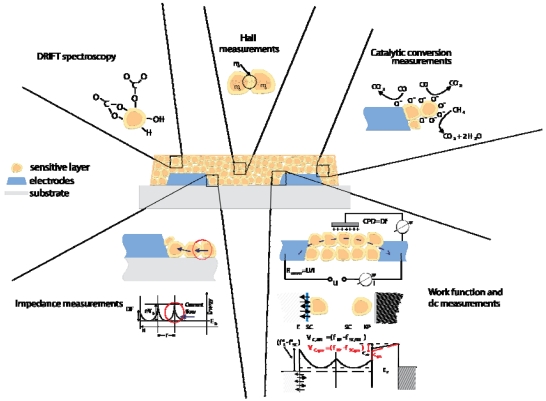
Operando methodology.

An overview is provided in [70]. The available measurement techniques are (see Figure 8):

- DC resistance measurements give information about changes in the concentration of free charge carriers induced by surface reactions.
- AC impedance spectroscopy allows for identifying charge depleted regions such as surface space charge layers or metal-metal oxide contacts and of the nature of free charge carriers (ions or electrons). The changes induced by gas reactions allow following the way in which charge transfer processes effect the free charge carrier transport and the dielectric properties.
- Hall effect measurements give information about the various contribution to the conductance changes (of mobility and/or free charge carrier concentration) when combined with the DC resistance measurements. They provide insights that help to model the conduction processes in the sensing layer.
- Work function changes are measured by the Kelvin method (details see Section 4). They help to evaluate the impact of surface reactions (charge transfer processes between gases and metal oxides). In combination with conductance measurements localized chemisorption and ionosorption can be discriminated.
- On-line gas analysis of the composition of the sensor ambient atmosphere allows determining the end products of solid-gas interaction and gives insight about the possible reaction paths.
- Diffuse Reflection Infrared Fourier Transformed Spectroscopy (DRIFTS) measurements are allowing for the identification of the adsorbed surface species involved in the gas solid reaction. It is one of the few spectroscopic techniques possible to be applied in-operando and its input is essential for the identification of reaction mechanisms.

##### Overview of operando results

The full battery of techniques was applied to the understanding of the influence of water vapor on the CO sensing mechanism in Pd-doped SnO_2_ [68] in combination with the modeling of the transduction processes in metal oxide layers [69]. This model system was chosen due to its relevance for application and due to the fact that the effects recorded with this material were significantly out of noise. By monitoring the catalytic conversion of CO to CO_2_ the adsorption/reaction of CO to CO_2_ at the surface of Pd-doped SnO_2_ was found to be a linear process. This indicates that the nonlinear sensor response is related to the transduction mechanism [71]. Also it was revealed that differently bound water and specific surface OH-groups react with CO. The so formed surface carbonate ions dissociate due to acidic intermediate products [72,73]. The DRIFTS results permitted to identify the generation of hydrated protons as being responsible for the increasing sensor conductance. This was crucial since the results from DC conductance, from AC impedance spectroscopy, and from work function measurements could not point at the responsible surface species. An especially important insight revealed by AC impedance spectroscopy was the identification of differently active regions in the sensing layer suggesting a catalytic effect of the Pt electrodes.

Differently prepared SnO_2_ materials (Flame Spray Pyrolysis powders [74]) were studied by a combination of work function and DC conductance measurements. The study of the interaction with oxygen indicated that for low oxygen concentrations at high temperatures (400 °C) the ionic oxygen species dominate whereas at lower temperatures (200 °C) a dipolar interaction of molecular oxygen with SnO_2_ has to be taken into consideration. This result questions the widely accepted mechanism for the interaction between atmospheric oxygen and SnO_2_, which takes into consideration only ionic adsorbed oxygen species. It can be combined with simultaneous DRIFTS and DC conductance measurements [75]. On hydrothermally prepared SnO_2_ a significant interaction between adsorbed oxygen ions and water vapor was observed which leads to the formation of terminal hydroxyl groups on tin dioxide surface. This observation is an evidence of water–oxygen interaction and could explain the observed change of electronic affinity during oxygen exposure by relating it to the build-up of new hydroxyl groups.

**Figure 9.**
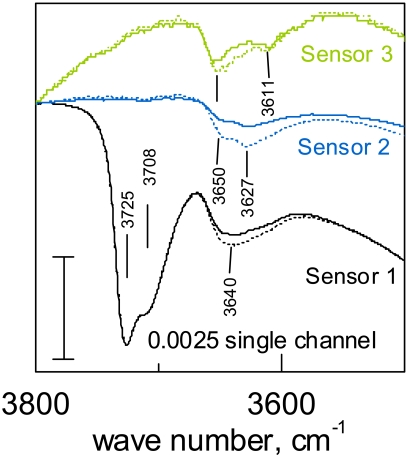
Single channel DRIFT spectra of different tin dioxide sensors exposed to oxygen at constant humidity level (3 ppm) 70 ppm O_2_ (drawn); 50,000 ppm (dotted). All sensors were screen-printed starting from pre-processed powders realized either by Flame Spray Pyrolysis [76] (Sensor 3) or by sol-gel (Sensor 1 and 2; with different calcinations temperatures, 500 °C and 700 °C, respectively).

Figure 9 shows results obtained for differently prepared undoped SnO_2_ materials (in fact, no surface sensitizers like Pd or Pt were added to the materials). The presented spectral region corresponds to the hydroxyl groups. It important to notice that, even if the surfaces of the different sensors are very different from the point of view of adsorbed species, the effect of oxygen exposure is very specific in all three cases. Obviously, the observed phenomenon has a general character for undoped SnO_2_. The interaction between water and oxygen explains also why the sensor effect for reducing gases in undoped tin oxide decreases in the presence of humidity.

Besides oxygen, water vapor and CO interaction with SnO_2_, hydrocarbon surface reactions were also investigated by DC conductance, catalytic conversion and DRIFTS measurements [77,78]. In all cases, the resulting products of the hydrocarbons interaction are CO_2_ and H_2_O, indicating a complete conversion. During propane interaction with SnO_2_, the surface ionic species plays a crucial role. The results indicate that the C_3_H_8_ dehydrogenation is initiated by hydrogen abstraction over a surface acid-base lattice pair site and leads to the formation of C_3_H_7_. Its further transformation is caused by reaction with the adsorbed oxygen species. The main intermediate surface species during the consecutive conversion of propane are ionic carbonates and carboxylates. Additional work function measurements confirm that the intermediates are ionic, i.e., they really stem from a reaction involving adsorbed oxygen species and not surface lattice oxygen.

Other interesting findings deal with the effect of the electrode nature on the gas sensing mechanism [79,80]. It is known that, e.g. Pt or Au electrodes, have an effect on the sensor response. Many speculations on the reasons exist. By using DC conductance and DRIFTS measurements it was demonstrated that the different nature of the electrodes changes the chemistry involved in CO sensing. Different distribution between carbonates and carboxylates, which are the reaction intermediates when CO is converted to CO_2_, were identified.

Other materials were investigated besides SnO_2_ (In_2_O_3_, WO_3_, TiO_2_ and Cr_2_O_3_). Very interesting is the identification of the origin of the transition in the conduction type of α-Fe_2_O_3_ from *n*- to *p*-type due to the presence of oxygen. This *n*- to *p*-type transition has an electronic origin [81]. If the resistance goes through a maximum when exposed to reducing gases, the work function decreases (decrease of the energy band bending). This rules out a change in the surface chemistry associated with the reaction with the reducing gases. The adsorption of oxygen leads to a strong depletion of electrons near the surface (upwards energy bands bending) and thus to the formation of a surface layer in which the conduction switches from *n* to *p*-type (inversion layer). This means that already the adsorbed oxygen traps electrons from the valence band, and holes as free charge carriers can appear. As long as hole conduction dominates, the resistance increases when the sensor is exposed to reducing gases, because the decrease of the hole concentration is not compensated by an increasing electron concentration. When the concentration of the reducing gases is high enough, the *n*-type conduction is restored and an increase of the reducing gases concentration determines a decrease of the sensor resistance. The overall impact of reducing gases is an initial resistance increase up to a maximum resistance value (corresponding to the intrinsic conduction regime) and a subsequent resistance decrease. This gets reversed by adding strong reducing gases, because the latter react with ionosorbed oxygen under release of electrons into the conduction band.

Recently, it was even investigated, how surface reactions-induced electrical changes are affecting the sensor signals of thick porous layers of p-type Cr_2_O_3_, by using simultaneous DC, work function changes (Kelvin probe method), and AC impedance spectroscopy measurements [82]. Based on them, a conduction model, which qualitatively explains the experimental data, was developed. It explains the large mismatch between the work function changes and the resistance change.

#### 3.1.2. One Dimensional Materials

The group of Mathur at the University of Cologne was very active in the last period in the field of anisotropic metal oxide nanostructures, especially SnO_2_, see e.g. In [83] the authors examined a general strategy for the fabrication and characterization of portable nanowires-based devices while in [84] they tried to take on the basic interaction between oxygen and the tin oxide nanowires. The synthesis of the nanowires is realized by catalyst supported chemical vapor deposition and their contacts are realized by using lithographic processes. The main idea is that by studying individual nanowires one can get a better insight in the sensing because the nanowires are a very good surface controlled single crystal and one avoids the complications of complex interconnections present in the classical thick or thin film approaches.

An alternative approach for the synthesis of nanowires was undertaken by Polleux *et al.* [85], namely a soft chemistry route. The resulting materials are crystalline WO_3_ nanowires self assembled into bundles at low temperature, which were drop coated onto alumina substrates. The resulting sensors were proven to show high sensitivity towards NO_2_.

### 3.2. P-type Materials

As discussed in the previous subsection, the *n*-type conducting oxide ceramics, in particular doped or coated SnO_2_, have received much attention in conductometric gas sensing. Their *p*-type conducting counterparts are scarcely discussed, although early publications already state the potential advantages of *p*-type oxide ceramics due to their stability [86,87] and their higher catalytic activity for oxidation reactions [88]. Some exceptions include the commercially available ammonia gas sensors, Cr_2-x_Ti_x_O_3_ [89,90], and NO_x_ sensors based on perovskite rare-earth metal oxides of the LnFeO_3_ family, where Ln is La, Nd, Sm, Gd and Dy [91].

In the 1990s, Moos *et al.* studied the sensor characteristics of SrTi_1-_*_x_*Fe*_x_*O_3-δ_, a *p*-type conducting semiconductor formulation with the perovskite structure [49,92]. Initially, this material was introduced for conductometric oxygen sensing at temperatures between 700 °C and 900 °C. Compared to the conventional potentiometric oxygen probes based on yttria-doped ZrO_2_ (confer Section 1), such conductometric sensors present a simpler set-up and are thus an inexpensive alternative when manufactured in thick-film technology [92]. As an additional advantage, a temperature independent resistance over this temperature range was reported for the SrTi_0.65_Fe_0.35_O_3-δ_-composition in particular [49,93]. Detailed studies were aimed at resolving the observed proneness of the devices to sulfur poisoning [94,95]. In other work by German researchers, *p*-type members of the system La_2_CuO_4_:LaFeO_3_ were identified as alternative candidates for temperature independent oxygen sensors [96-98] and a model of the sensor behavior was proposed [99].

**Figure 10.**
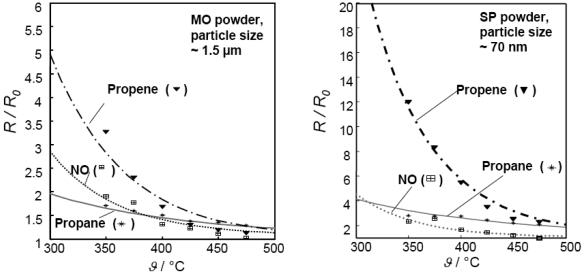
Comparison between model (lines) and experimental results (symbols). (a) Coarse-grained powder, μm scale. (b) Fine sol-precipitated powder, nm-scale (from [103]). Reproduced by permission of the PCCP Owner Societies.

In addition to the promising oxygen sensor characteristics of SrTi_0.65_Fe_0.35_O_3-δ_, the members of the SrTi_1-_*_x_*Fe*_x_*O_3-δ_ perovskite family with *x* ≤ 0.2 have been reported as promising materials for hydrocarbon sensing in the temperature range from 350 °C to 450 °C [100,101]. The initially studied screen-printed sensor elements presented a fast, stable, and reproducible hydrocarbon response. In order to improve sensor functionality and to identify corresponding key parameters, the material characteristics were optimized with respect to operating temperature, iron content, film thickness, and particle size. By enhancing the surface-to-volume ratio of the devices, which was achieved either by the use of nano-scaled sol-precipitated precursors [50] or by electrospinning techniques (cf Section 3.4) [102], the selectivity towards propene was increased. To reflect the difference between this *p*-type material and the commonly used *n*-type semiconductors of the Taguchi type (cf. Section 3.1), a novel sensor model was proposed to explain the sensor effect in a quantitative way [103]. For *n*-type sensor models, the redox process that is responsible for the sensor response to reducing gases is confined to the sensor surface. According to observations reported for *p*-type semiconducting oxidation catalysts, however, it is very likely that lattice oxygen is exchanged when they are exposed to reducing gases [104]. Hence, a reduction process affecting the entire bulk was assumed to govern gas sensitivity of SrTi_1-x_Fe_x_O_3-δ_ films in the refined *p*-type model. Figure 10 illustrates the good agreement between the model results (lines) and the experimental data (symbols) [103]. Here, the conductometric sensor response of two sensors with a different microstructure towards a variety of gases was considered.

### 3.3. Zeolites

The versatile materials class of zeolites (Class of aluminosilicates with the general sum formula ${\text{Me}}_{\text{y}/\text{m}}^{\text{m}+}\left\lfloor {\left({\text{SiO}}_{2}\right)}_{\text{x}}\cdot {\left({\text{AlO}}_{2}^{-}\right)}_{\text{y}}\right\rfloor \cdot {\text{zH}}_{2}\text{O}$ [105]. The tetrahedral silica and alumina building blocks form a 3-dimensional framework, in which cations Me^m+^ compensate the residual negative charge caused by the alumina atoms.) with a 3D-framework, ion-exchange capacity, and/or catalytic activity has received increasing attention in various applications. Due to their unique property spectrum, zeolites are of particular interest in the field of gas sensing [106-108]. In a large portion of the corresponding literature, these materials figure as a mere auxiliary phase. They are applied for example as a filter to prevent cross-interfering species from reaching the sensor surface or as an inert matrix to encapsulate the gas-sensitive agent e.g. [109-112]. However, research work in Germany has recently focused on devices where the zeolite itself acts as the sensitive element.

After studying the effect of water vapor and polar organic molecules on zeolite conductivity in the temperature range from 40 °C to 110 °C, several German groups proposed (Guth *et al.*, Fetting *et al.* and Plog *et al.*) coworkers were the first to propose a corresponding conductometric sensor concept for humidity and combustible gases [113-116]. Some years later, Simon and co-workers proposed an impedimetric zeolite-based sensor for humidity detection (400 °C ≤ *T* ≤ 600 °C) in harsh environments [117]. Here, the impedance of a Si-rich zeolite film was found to respond linearly and reversibly even to traces of humidity. Assuming an adsorption-desorption equilibrium of water molecules in the zeolite framework, the effect was attributed to a solvate supported proton transport.

Earlier results by the Simon group provided the basis for the development of a highly selective zeolitic ammonia sensor [118]. While studying the effect of ammonia on dehydrated zeolite BEA, they observed an increase in the protonic bulk conductivity of the material. These findings were attributed to the adsorption of NH_3_ molecules, which support the proton transport from one aluminum site within the zeolite framework to the next [119]. Since the effect depends essentially on the amount of adsorbed NH_3_, i.e., on the ammonia partial pressure *pNH_3_*, proton-conducting zeolites are promising candidates for conductometric or impedimetric ammonia sensors. The robust impedimetric zeolite sensor element discussed in [120] was intended for automotive exhaust gas applications and presented no significant cross-interference of hydrocarbons, CO_2_, CO, and O_2_ when operated at 420 °C.

In later work [121], Simon *et al.* discussed a similar sensor concept using proton-conducting zeolite MFI for conductometric amine detection. Also in this case, reversible and concentration dependent changes of conductivity with a fast response time were observed. However, a refined model of interaction of the amines with the mobile protons of the zeolite remained unsolved.

The Moos group reported a novel ammonia sensor using Fe-loaded zeolites of the framework type MFI [122]. This material is known as a highly active candidate for the selective catalytic reduction (SCR) of NO_x_ with ammonia, a catalyst concept discussed for automotive exhaust gas aftertreatment [123]. The high-temperature stable zeolite was applied directly as the functional sensor film. By measuring its impedance at 500 °C, selective ammonia sensor elements were obtained. At this temperature, only comparatively high concentrations of NO_2_ and propane caused a small cross-interference whereas the NO sensitivity was negligible. The same group also used the same zeolite without Fe-doping to prepare a mixed-potential ammonia sensor for automotive applications [6]. The system MFI, Au / YSZ / Au presented a high ammonia sensitivity with the additional advantage to optimize independently the electrode (Au) and the zeolite catalyst (MFI) layer.

In addition to bulk effects within the zeolite framework, interface effects between the zeolite and an adjacent material may cause a sensor response. Hagen *et al.* studied this effect by comparing Pt-loaded zeolite MFI/Cr_2_O_3_/Au to Pt-loaded zeolite MFI/Au [124] and by using four-probe impedance spectroscopy [125]. They showed that the sensor response to hydrocarbons, i.e., a pronounced increase of the low frequency impedance, only occurs in the presence of the metal oxide interfacial layer between the zeolite and the electrodes. If the zeolite/Cr_2_O_3_ interface is missing, the sensor device looses functionality. This discovery led to the successful transfer of the sensor concept from thin film processing to more cost-effective film technology without using PVD or vacuum processes [126]. A very selective sensor element was prepared, which was almost insensitive toward H_2_, CO, NO, CO_2_ and O_2_ (if oxygen is available in excess). In recent work, Fischerauer *et al.* discussed a mechanistic model of the sensing mechanism [127] taking into account the ionic conductivity of the zeolite and the p-type semiconductor properties of Cr_2_O_3_. In addition, the blocking electrode characteristics of the zeolite/Cr_2_O_3_ interface were included. By this model, the charge carrier density in the Cr_2_O_3_ film was identified as a crucial parameter influencing the sensor current. Since the ambient hydrocarbon concentration modulates this density due to conventional semiconductor-gas interactions (confer Section 3.1), the sensor current and hence the impedance spectra respond to concentration changes.

It should be noted here that gas sensor research is just at the outset of possibilities arising from utilizing framework structures materials. This topic is not restricted to inorganic materials with channels and pores but may be greatly enlarged to organic materials. One example shall complete this section. Very recently, metal-organic framework (MOF) materials were investigated as impedimetric gas sensor materials in the range 120 °C - 240 °C. They show very reproducible responses to water [128] without being affected by O_2_, CO_2_, C_3_H_8_, NO, or H_2_.

### 3.4. Further Approaches for Selectivity Enhancement

A newly reported promising way for the improvement of the sensing capability of semiconducting metal oxides is to keep the base chemistry of the materials as it is and to work on “physical” methods to get to new sensitivities. Important improvements in this field came from Kohl *et al.* (University of Gießen). One relates to the notion that many oxides at elevated temperatures are mixed conductors. The usage of a polarizing electrical field during the measurement leads to a movement of bulk donors and results in changes of the surface charge. Instabilities as well as changes of sensitivity may occur due to that and are described in models. This leads to general considerations about improving the result by a careful selection of the dynamic measurement procedure [129]. The other approach imposes nanosized surface corrugations on materials with known chemistry. These mesoporous materials are created by structure replication e.g. hexagonal mesoporous silica variants. The sensitivity here obviously depends not only on the surface-to-volume ratio, but also on the nanoscopic structural properties of the base materials [130,131].

The University of Hamburg and Sony Research, Stuttgart (Vossmeyer) pursue a novel way of conductometric readout of non-oxidic materials that is targeted to a new generation of low-power / room temperature operated sensors [132]. They use nanoparticles from noble metals that are embedded in a cross-linked way in dendrimetric organic materials. This matrix stabilizes the nanoparticles. Readout can be done mass sensitive (quartz microbalances) as well as conductometric by the application of such a film on an interdigitated electrode structure. With conductometric readout the resistances changes to gas exposures are relatively small (below 1 % relative change) but appear to be exploitable due to a low S/N [133,134] The aim is to arrive at a selective adsorption of guest molecules from the gas phase

### 3.5. Novel Deposition Techniques

Driven by the growing need for high performance gas sensors, a large number of innovative deposition techniques have been introduced and investigated. Compared to standard thick and thin film processes, the novel methods either present with direct-write features or offer precise control of micro- and nano-structural features of the deposited materials [135].

Among the most cost-efficient novel techniques feature various types of suspension-free spray deposition. Since no solvent needs to evaporate, these techniques present a major advantage over the wet sol-gel based coating methods. Hence, the films are more homogeneous and less prone to cracking. At the University of Tübingen [76], flame spray pyrolysis was studied for gas sensor preparation (results see Section 3.1). This versatile and effective technique allows to produce metal oxide powders and to deposit thin films with the possibility to control film morphology and powder particle size in the nm range. After formation in the flame by nucleation, coagulation, and coalescence, nanoparticles undergo direct (in situ) deposition onto suitable sensor substrates. A very fast and clean single step process for sensor preparation is thereby obtained. The resulting thick films are highly porous and present a large accessible surface, which makes flame spray pyrolysis a promising candidate for rapid and low cost sensor production. Quality and properties of the films and powders depend on the process parameters. In [136], highly porous pure or Pt-doped SnO_2_ powders were prepared by direct flame spray synthesis. The synthesized powders were screen-printed and annealed at temperatures up to 500 °C. With these devices, CO concentrations well below 10 ppm were detected. Functional nanoparticles were directly deposited from the aerosol phase onto standard sensor substrates forming highly porous films [137]. By repeating layer deposition, multilayer structures were prepared consisting of doped and undoped gas sensitive SnO_2_ and Al_2_O_3_ as a filter layer.

**Figure 11.**
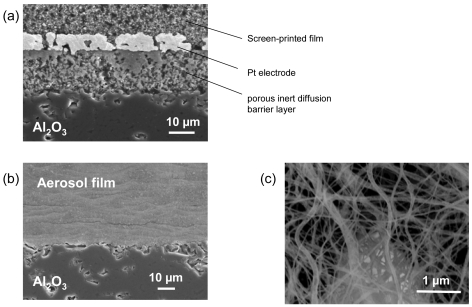
One sensing material, different microstructures: (a) Porous SrTi_1-x_Fe_x_O_3-δ_ film prepared by conventional screen-printing. (b) Aerosol-deposited compact layer. (c) SrTi_1-x_Fe_x_O_3-δ_ fiber structures obtained by electrospinning.

As another spray method, the aerosol deposition technique has recently been investigated for gas sensor preparation at University of Bayreuth [138]. This low-temperature technique was first proposed in 1999 as a simple method for the preparation of dense piezoelectric films at room temperature [139]. In a continuously shaken aerosol generator, the precursor powder is fluidized. Using a vacuum pump, the carrier gas stream loaded with powder particles is then accelerated towards the substrate. If the various process parameters, e.g. powder particle size, flow rate, or vacuum conditions, are carefully adjusted, the impact energy is sufficient to prepare a dense layer on the substrate without the need for a sintering step at high temperatures. For some materials, this low-temperature deposition process might simplify the sensor preparation to a large extent. One example is the SrTi_1-x_Fe_x_O_3-δ_ perovskite system with its promising temperature-independent oxygen sensor characteristics (cf. Section 3.2 for details). When prepared by the conventional screen-printing technique, SrTi_1-x_Fe_x_O_3-δ_ films present a stability problem during sintering at 1,200 °C when in direct contact with alumina. In an initial feasibility study, SrTi_0.7_Fe_0.3_O_3-δ_ sensor films were prepared at room-temperature by low-temperature aerosol deposition. The spray-deposited films presented a dense and homogenous microstructure (confer Figure 11b) with an excellent adhesion to the substrate. The oxygen sensing behavior of the films was assessed and found to be in perfect agreement with earlier literature data on this material [49].

Due to the high surface-to-volume ratios achieved by electrospinning, this novel deposition method is a very promising tool for the preparation of conductometric sensor elements. Its operational principle relies on electric forces used during deposition. A high voltage, in the kV range, is applied between a substrate and the needle of a syringe, which contains the precursor solution and is operated by a programmable syringe pump to achieve a constant delivery rate. The precursor solution is prepared by adding an appropriate polymer solution to the unreacted solvated components of the sensor material. An important feature is the choice of an effective, highly volatile solvent, which evaporates in-situ. Appropriate systems are polyvinylpyrollidone (PVP) in ethanol or cellulose acetate (CA) in acetone. Under the action of the electric field, a fine polymer fiber network forms, which encapsulates the metal oxide precursor. During a subsequent thermal treatment, the polymer matrix is removed, and ceramic nano-wires remain on the substrate. In a collaborative study, researchers at University of Bayreuth investigated this technique for the preparation of fiber networks of ternary SrTi_1-x_Fe_x_O_3-δ_ perovskites as shown in Figure 11c. The hydrocarbon sensor characteristics of the electrospun fibers were superior to those observed with conventional devices prepared by screen-printing of microscaled powders [102].

In addition, the Simon group at RWTH Aachen and the Maier group at University of the Saarland directed considerable research effort to high-throughput methods for screening gas sensor materials. These methods, originally developed for pharmaceutical purposes, allow investigation of a multiplicity of materials prepared in the form of “libraries”. Compared to the conventional ‘one at a time’ strategy, this accelerates material synthesis and characterization. In [140], ZnO and In_2_O_3_ nanoparticle films were prepared on multi-electrode substrates using a laboratory roboting unit and functionalized with various noble metals by surface doping. High throughput impedance spectroscopy was used to identify the most suitable candidates for NO sensing. A similar technique was used in [141,142] to screen the gas sensing properties of a large variety of semiconducting metal oxide materials. An overview of the modus operandi is given in [143]. Besides the rapid identification of suitable gas sensor materials, the groups aim at the understanding of composition-to-property relations in semiconducting metal oxide nanoparticles.

## 4. Field Effect Sensors

Another very promising technique for low cost/low power gas sensors is the work function readout via field effect devices, which was first introduced with the H_2_-sensitive field effect transistors with heated Pd-gate [144]. In this case, H_2_ diffusing through the Pd-gate applied directly on the channel isolation of a FET-structure produces a potential at the Pd/channel insulation interface that modulates the drain-source current. Also called “Lundström-FET”, this approach is somewhat limited in terms of detectable gases. However, it triggered research on a multitude of sensor variants. They directly use an electrical potential arising due to gas adsorption at the sensitive material. This gas adsorption at the surface leads to what physicists call a change in the work function and chemists a change in the electro-chemical potential. Obviously it is quite easy to control since it directly relates to surface properties and is not affected by the multitude of effects of electrical currents crossing a solid state sensor device.

### 4.1. Device Technology

**Figure 12.**
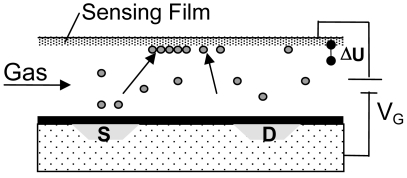
Scheme of a suspended gate GasFET. The gate electrode is suspended and covered with a gas sensitive layer. The electrical potential generated by gas adsorption acts as an additional gate voltage and changes the source-drain current. Reprinted from [150] with permission from Elsevier.

The size of these surface potential changes is in the order of 100 mV at suited sensitive materials and thus it can be used to drive a FET device. A sketch of the setup is shown in Figure 12. The insulation of the transistor channel has to be made of a chemically inert material to avoid additional gas reactions at this surface; otherwise an additional potential change occurs there. The usage of LPCVD deposited Si_3_N_4_ has shown to be a good choice for that.

**Figure 13.**
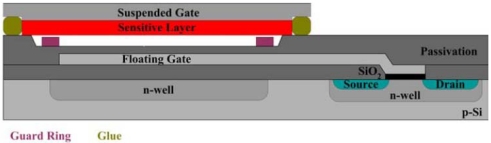
Schematic cross section of a floating gate (FGFET) type transducer that improves the coupling of the work function voltage to the FET. The capacitance well electrode can be additionally used to set the optimal working point in the transistor characteristics.

Besides Janata [145], the pioneers in the construction of such type of set-up were Eisele and Doll [146,147], who used a hybrid approach with one part being a gateless FET done with conventional Si-technology and the other a substrate equipped with a sensing film, both brought together to form a gas sensor. The weakness of such a device is that due to the small capacity of the air gap, only a small portion of the generated potential is actually driving the FET. Following work from Gergintschew [148], several options have been introduced to make more efficient use of the generated voltage. One significant improvement consists of the formation of a larger area capacitor build from the suspended gate and a floating gate. The floating gate then transmits the potential coming from the gas sensitive layer to a small FET-device with a short channel [149]. This basically minimizes the loss of sensing signal due to weak coupling via the air gap (Figure 13).

**Figure 14.**
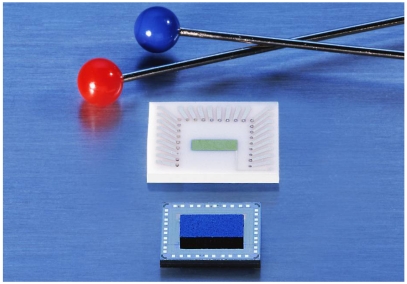
Actual state of the art of a GasFET: the gate part carrying the sensitive layers is glued to the silicon chip (below). The white ceramic part is a carrier that might be used to host the small chip. Attaching the sensitive layer to this ceramic constitutes an alternative construction of the device.

For industrialization, a flip-chip variant of this device was designed, allowing easy mounting, a precise definition of the air gap, and low-processing temperatures [150]. The base idea for the formation of the air gap here is to have a channel in the Si and to mount the gate chip with a large flat surface area of the sensing layer in direct contact with the chip surface. The current state of this sensing technology is depicted in Figure 14:

- An appropriate sensing material is deposited on a flat carrier substrate forming what eventually becomes the gate-electrode. The preparation conditions are not limited by any Si-electronics related constraints.
- The Si FET-chip is separately prepared in standard CMOS. Electronics for driving the sensor may be integrated in the Si-Chip
- Finally both parts are bonded together so that a defined air gap is formed.

Using an open gate FET in a setup like in Figure 14, the change in work function can be directly measured with a small device. When four FET-devices are included in the Si-Chip, they can be combined with four different sensing materials. Hence, a very compact sensor array can be constructed [151]. Alternatively, FET channels can be used to compensate for the influence of varying ambient temperature on the FET itself, or special temperature independent operation conditions of single transistors (“isothermal point” of the transistor characteristics) are chosen.

These sensors are characterized by an unprecedented freedom in the choice of sensing materials. The gas receptor does not need to be an oxide. Organic molecules, polymers, metals, or salts may be used in the sensing platform. Since the sensors can be operated at room temperature, heating is not required. Multiple readout channels allowing for tiny sensing arrays can be realized on one single chip.

The base potential of this sensing technology arises from the fact that the measurand comes from the direct measurement of surface effects. One consequence is that sensing materials in these devices do not need to be a semiconductor: metallic conductors or insulating materials can be used as well.

### 4.2. Gas Sensing Materials for Suspended Gate FETS

**Figure 15.**
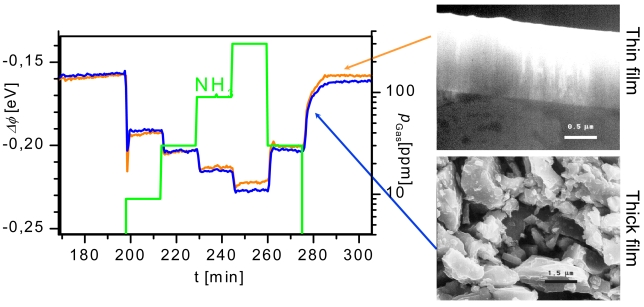
Work function change of two different preparations of TiN at room temperature in response to NH_3_ exposure in wet synthetic air measured with the Kelvin method. Reprinted from [152] with permission from Elsevier.

Similar to direct thermoelectric gas sensors (cf. Section 1.4), in contrast to classical semiconducting metal oxide gas sensors, the gas sensing properties do not depend on the morphology of the sensing materials. An example demonstrating this has been found during the investigation of gas-induced work function changes of TiN. TiN is quite inert in view of its chemical properties, but has been found to react mainly with NH_3_ and thus can be used as a selective material for NH_3_ detection in environmental air. The preparation of TiN as a sputter-deposited compact thin film and as screen printed porous thick film resulted in the same gas sensing properties due to the fact that mainly the TiN surface determines the gas sensing characteristics [152,153], see Figure 15. Since the device is working at room temperature, no heating power is required. The TiN-based sensor serves also as a nice example for a sensing material with metallic conductivity.

Several noble metals showing a stable surface configuration are applied detection layers. They comprise noble metals like Pt [154] or Pd [155] for H_2_ detection. Especially with the adsorption of H_2_ on Pt, amazingly high surface potential changes of 500 mV and more are observed. The reason for this can be seen in the high reactivity of Pt to H_2_ even at room temperatures. The problem with high sensitivities is that the strong reaction with H_2_ implies a strong influence on the “natural” adsorbates on these sensing layers. In this case, it is a removal of the adsorbed oxygen on the Pt surface, which is reconstituted only after long periods, thus causing some hysteresis effects after repeated strong H_2_ expositions. This leads to a new operation strategy for the reactive Pt, which is further coated with a gas diffusion filter [156]. Now, the differences in the diffusion of O_2_ and H_2_ cause the major sensing effect, significantly improving the reproducibility of the sensor readings for H_2_.

Au is another example for a stable metallic sensor materials, which is suitable for the detection of strongly oxidizing gases like NO_2_ or O_3_ [157]. Semi noble metals like Ag are reported to be sensitive to H_2_S and Cl_2_ [158].

This leads to the metal oxides that form a large group of sensing layers suited for GasFETs. In contrast to the heated semiconducting metal oxides, which are a broad band sensor for reducing or oxidizing gases, metal oxides at room temperature do not show a significant reaction to the reducing capability of gases. This is demonstrated by an absence of a sensor effect to small reactive hydrocarbons using polycrystalline layers of porous Ga_2_O_3_ and work function readout. Such a layer, however, is reactive to solvents that couple to the metal oxide through their functional groups [159].

**Figure 17.**
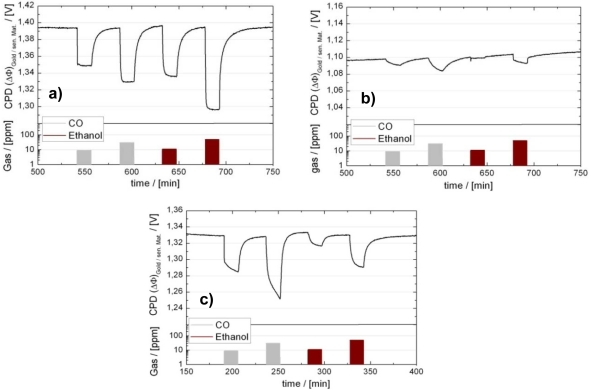
Work function change of the Ga_2_O_3_/Pt system to a reducing gas (CO) and ethanol at room temperature in wet synthetic air: (a) Immediately after preparation (last thermal budget 700 °C for 2 h to form nano-dispersed Pt. (b) After 800 h storage at room temperature. (c) After storage and thermal activation at 175 °C for 5 mins. The thermal activation worked repeatedly during all experiments.

Using films of metal oxides that carry a catalyst dispersion, a reactivity to the reducing character of gases can be obtained even at room temperature, when a thermal activation was done. Figure 17 illustrates this for a nanosized catalyst dispersion supported on a polycrystalline Ga_2_O_3_ sensing layer. This layer is developed to create a room temperatures-operated TVOC (total volatile organic compounds) sensor. Without activation, the sensitivity is almost completely lost. Using an intermittent thermal activation at moderate temperatures (< 200 °C in wet room air that might be done one time daily for a few minutes), a sensitivity for all tested reducing gases can be maintained, even when the sensing material is operated at room temperature. Obviously some reactive oxygen species are formed during the thermal activation. They are the stored in the sensing material [160]. The oxide enhances the lifetime of the activated species. Using only Pt as sensing layer, the gas sensitivity declines after a few hours after thermal activation. Using the Pt/Ga_2_O_3_ systems, the gas sensitivity is preserved at least one order of magnitude longer. Sensitivity to all gas groups relevant for indoor air smell monitoring can be achieved with a proper combination of finely dispersed Pt and thermal activation [160].

**Figure 18.**
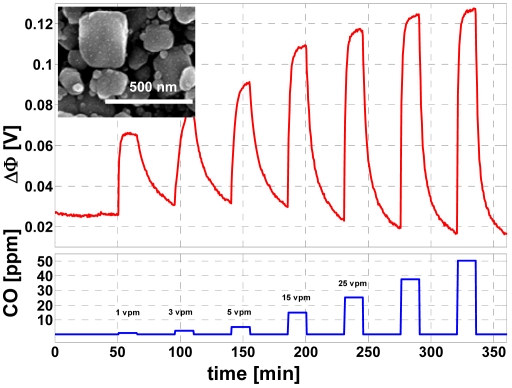
Sensing characteristics towards CO in ambient air of a GasFET equipped with a polycrystalline sensing layer of Pd-doped SnO_2_. In the inset, the size of SnO_2_ crystallites with the impregnated Pd clusters are shown. Measurements were conducted at room temperature after a preceding thermal activation. Reprinted from [162] with permission from Elsevier.

Also the classical SnO_2_/Pd system can be used to detect CO (Figure 18). According to the model with the formation of some reactive oxygen species at the surface, it needs some intermittent thermal activation. Then, CO can be detected even at room temperature with the SnO_2_/Pd system [161,162]. Selectivity to interfering gases is roughly comparable to the classical heated conductometric semiconducting SnO_2_/Pd sensors.

**Figure 19.**
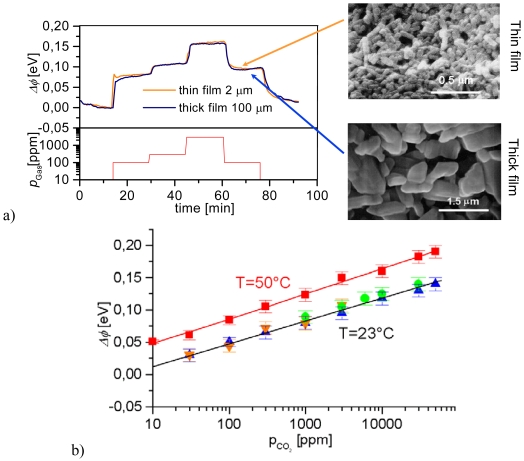
Detection of CO_2_ with the BaCO_3_ system: (a) Work function change of two different preparations of BaCO_3_ at room temperature in response to CO_2_. (b) Sensor characteristics for CO_2_ detection in wet synthetic air. Reprinted from [163] with permission from Elsevier.

Even sensors for gases that are hard to detect with semiconducting metal oxide gas sensors could have been implemented due to the versatility in the chemical structure of the sensitive layer. In one example, the base idea was to work with carbonate-based sensing layers, since they show some chemical similarity to CO_2_. The best material found in this system was BaCO_3_. It appeared to be a reversible sensing material for CO_2_ even at ambient temperature [163]. Again, the sensitivity is independent on the morphology (Figure 19a). Thin films prepared by a colloidal suspension showed the same CO_2_ sensing characteristics like large-grained thick films. The slope of the sensor characteristics temperature independent within a certain range and obeys a logarithmic behavior (Figure 19b), which emerged to be typical for most work function based sensing materials over a wide concentration range.

One obvious question with the gas detection by surface reaction at ambient temperature is the role of water in the detection process. In atmospheres with humidity, always several monolayers of water exist on the surface. They might hinder the gas sensing process. Results by Kelvin probe investigations demonstrated that the presence of humidity is necessary. The discussed BaCO_3_ films completely lose their CO_2_ sensitivity in absolutely dry atmospheres. IR spectroscopic investigations have shown that the CO_2_ detection process is a reversible formation of dimeric HCO_3_^-^ in the water film on the surface [164]. These films are open porous, allowing gases to diffuse through the whole film and to reach the bottom electrode. Therefore, the electrode material has a significantly affects the cross sensitivity of the sensors [165].

Mixed oxide systems on the basis of BaTiO_3_/CuO [166] are also suitable for room temperature detection of CO_2_. The sensitivity is perhaps correlated with the formation of a surface phase of BaCO_3_. Following the approach of the acid-base reaction mechanism for the detection of CO_2_, even better results have been obtained with polymers that contain basic-type groups [167].

**Figure 20.**
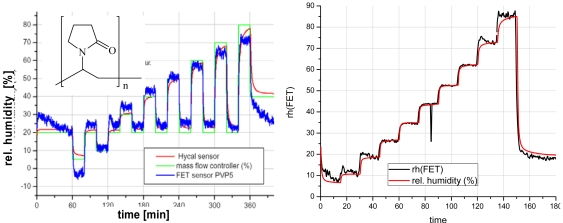
Polymers for humidity detection employed in the GasFET platform. Left: polyvinylpyrrolidone with classical work function readout. Right: polyamide readout by the gate pulse method. Both signals are compared to a standard capacitive humidity sensor.

The class of polymeric materials turned out to be extremely powerful for this type of sensors. Besides materials like polysilsesquioxane that appeared to be selective sensors for solvents, the classical humidity sensing polymers can be incorporated into the FET sensing platform. Figure 20 shows two examples of such materials for humidity sensing [168]. The usage of these materials in the GasFET sensing platform is twofold. One is the direct readout of surface potentials. On the other hand, an active readout strategy can be pursued. Then, a voltage pulse is applied to the gate electrode and the following transient reaction of the source-drain current is evaluated. This mimics the capacitive readout of the polymers in the classical humidity sensors.

### 4.3. Applications of GasFETs

One promising field for applications of such sensors is air monitoring in buildings. Due to their small dimensions and moderate costs, they can be used for distributed sensing networks allowing local sensing of air properties. Due to their low power consumption at ambient temperature operation, battery-operated sensing nodes that communicate wireless can be established. Since there are no high labor costs for wiring, an additional and decisive cost advantage for the sensor user occurs.

An important application of such distributed sensors will be the control of the of indoor air quality to allow for an on demand ventilation of different locations in a building. Good examples are meeting rooms. Usually, when the room is unused the ventilation is too high, and is much too low, when a meeting takes place. Similar situations occur at work places due to the changing occupancy and due to the varying loading of the air with contaminants. The real air quality will be determined by measuring

- Temperature and humidity. The relative humidity (r.h.) has to be kept optimally at a level between 40 - 60 % which is best for comfort as well as for the performance of people.
- The CO_2_-content: human breath enriches the air with CO_2_. At a level of 1,000 ppm, the first physiological reactions occur. Above 2,000 ppm people tend to become tired.
- The overall smell level. Smell arising from human sources as well as from building components has a distracting effect, lowering the comfort level as well as the effectiveness of people. Some components have direct unhealthy effects.

The detection of all these quantities is required to come to a proper view on how inhabitants will perceive the environment. Due to the array capability and the versatility of the GasFETs, it will be the first time that a moderate cost solid state gas sensor detects all influential factors in various places in a building. The local sensing of the air quality will then allow for a local adjustment of the ventilation. The benefit of such improved air conditioning systems will not only be significant energy savings but also an increase in comfort. Extending the capabilities of the sensor array by developing and adding additional layers will allow detecting a wide range of possibly hazardous situations as well.

**Figure 21.**
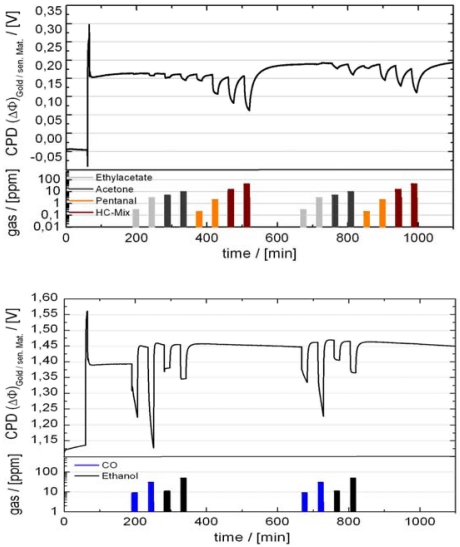
Detection of the lead gases for “smell” with a Ga_2_O_3_/Pt sensing layer with a room temperature-operated GasFET. To keep the sensitivity to the full gas spectrum, an intermittent thermal reactivation (e.g. once in the night for a few min) is necessary.

More complex than determining r.h. and CO_2_ is smell detection. An analysis of the indoor air with classical chemical analytical equipment like GC reveal the presence of hundreds of volatile organic compounds in indoor air with concentrations ranging from the sub-ppb to the ppm range. The development of receptor layers is not able to take this full range into account. The solution here is to group the gases into classes with similar chemical structure and reactivity to select a “lead-component” for the groups and to define a trigger level that is either adapted to the level of human smelling perception or to the maximum allowed exposure level. Typical classes are, e.g., aldehydes, alcohols, ketones, or unsaturated aliphatic hydrocarbons. All lead-components of these classes can be detected with a polycrystalline Ga_2_O_3_ sensing layer and a nano-dispersed Pt catalyst [160], see Figure 21.

Besides the classical use of gas sensors, an upcoming application field for gas sensors are medical applications. This relates to the analysis of human breath with the goal to detect marker gases that are characteristic for metabolic malfunctions or diseases. In classical Chinese medicine, physicians smell the odor of their patient's breath to obtain an indication for their health state. The analysis of exhaled breath by gas sensors offers the potential for minimal disruptive measurements for diagnosis or therapy control. Since the marker gas concentrations are usually very low and since they need to be selectively detected, only advanced and versatile gas sensors can be used for these purposes.

A valid medical example for this is bronchial asthma, where the biomarker NO is already well established [169]. An increase of the regular value of 10-30 ppb of NO in the human breath to a threshold of 100 ppm indicates pulmonary inflammation processes. For asthmatics, elevated NO has been validated as a reliable marker for the onset of an asthmatic crisis.

**Figure 22.**
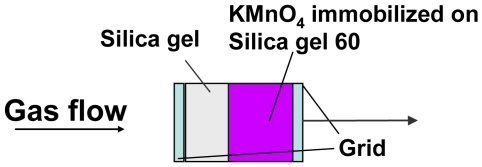
Schematics of the NO to NO_2_ converter for human breath analysis. The gas passes through a two stage filter that dehumidifies and performs the oxidative conversion.

With the GasFET technology it seems possible to develop sensing systems being sufficiently selective, sensitive, and stable. No proper sensing layer for NO has been realized up to now, however, sensing layers based on Cu-Phthalocyanine have shown the ability to selectively detect NO_2_ down to the ppb range [170]. An efficient NO to NO_2_ converter for human breath has been developed [171]. The base idea is to let pass the exhaled air through a two stage filter. The first stage consists of pure silica gel and removes excessive humidity from the breath. Silica gel which is impregnated with a strong oxidizing agent (KMnO_4_) to oxidize NO to NO_2_ is the second stage (Figure 22). Results obtained at a gas flow 2.5 l/min showed an NO to NO_2_ conversion rate of 95 % after the filter. Some loss of nitrous oxides due to adsorption on the filter has to be considered, but the actually obtained recovery of NO_2_ amounts to approx 90 % and is therefore sufficient for this application.

**Figure 23.**
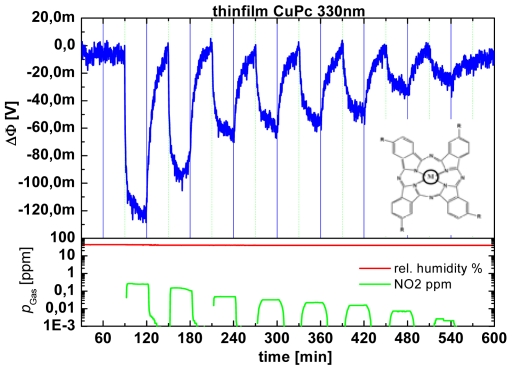
NO_2_-Sensing behavior of GasFETs with Cu-Phtalocyanine thin films: (a) Transient response of the work-function. (b) Sensing characteristics.

**Figure 24.**
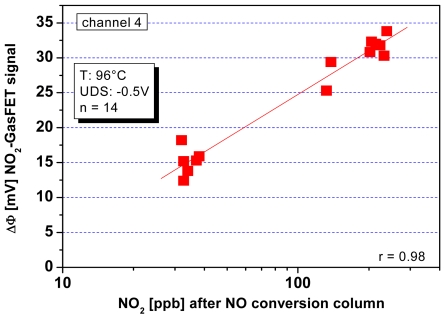
Results for the NO_2_ detection in simulated human breath using the NO to NO_2_ converter and a GasFET equipped with a Cu-Phthalocyanine sensing layer. The rise of the NO content from the normal value of up to 30 ppm to the threshold of 100 ppm can be clearly separated.

Sensors for selective detection of those low levels of NO_2_ are possible using the GasFET technology (Figure 23). They need to be heated to avoid possible condensation effects of water and sticking of NO_2_ on the walls and to respond sufficiently fast [172]. The results shown in Figure 24 demonstrate the ability of such systems to detect the increase of the NO concentration in human breath to the threshold of 100 ppb which needs to be detected. Since these sensing systems are moderate in costs, they have the potential to provide an in-home warning and therapy control device for asthmatics, a group of persons which is a significant part of the population in industrialized countries - with rising tendency.

### 4.4. Field Effect Sensors: Discussion and Outlook

The gas sensors based on suspended gate FETS are characterized by some interesting features:

- They are able to work at room temperature. This reduces the energy demand for operation and avoids thermal decomposition of instable gases at the sensing surface.
- Due to their functional principle they make direct use of surface properties, thus facilitating the preparation of materials with reproducible sensing properties.
- They enable the application of various classes of sensing materials. This enhances the chances to generate a sensing surface that has a surface chemistry that allows the direct and selective reaction with the target gas to be detected.

Arrays with these gas sensors tend to be available. In addition, it has to be stated that the potential for new sensing layers is far from being exhausted. Due to the possibilities to incorporate existing sensing layers known from other transducers and to use even new chemical affinity systems, various new sensors will come up that are based on this platform. One tendency is the research for selective sensing layers, e.g. ones assisted by a sterical selectivity mechanism like molecular imprinted polymers (MIPS), zeolites and the newly investigated mesoporous oxides [173]. The other tendency is research on new variants of the GasFET transducers that will allow operation temperatures above the normal Si-regime [174]. In the future, this might close the gap between high-temperature operated semiconducting metal oxides and work function devices.

## Conclusions

The present status report highlighted how gas sensor research prospers in Germany. Many types of sensors are under development for various applications. In addition to step-by-step improvements of conventional principles, e.g. by the application of novel materials or by understanding-driven tailoring of the sensor films, novel principles turned out to open new markets. In the field of high-temperature gas sensing, real-time in-situ diagnosis of chemical reactions, for example in combustion processes or in exhaust gas aftertreatment, is an increasing application field, in particular for potentiometric or conductometric principles. With respect to low-temperature gas sensing, novel field effect devices present a high potential to enter the market for indoor air control or medical applications.

## Figures and Tables

**Figure 16.**
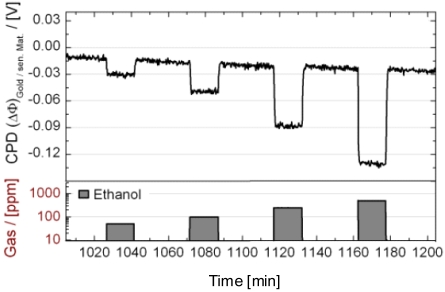
Reaction of a porous Ga_2_O_3_ thick film at room temperature in wet synthetic air to ethanol. No signal to reactive small hydrocarbons (ethane, ethene, ethine, propane mixture) in the range of 1,000 ppm is observed.

## References

[1] Yamazoe N. (2005). Toward innovations of gas sensor technology. Sens. Actuat. B-Chem..

[2] Williams D.E. (1999). Semiconducting oxides as gas-sensitive resistors. Sens. Actuat. B-Chem..

[3] Chen Z., Lu C. (2005). Humidity sensors: a review of materials and mechanisms. Sens. Lett..

[4] Moos R. (2005). A brief overview on automotive exhaust gas sensors based on electroceramics. Int. J. Appl. Ceram. Technol..

[5] Janke D. (1981). A new immersion sensor for the rapid electrochemical determination of dissolved oxygen in metallic melts. Solid State Ionics.

[6] Moos R., Schönauer D. (2008). Review: recent developments in the field of automotive exhaust gas ammonia sensing. Sens. Lett..

[7] Zosel J., Müller R., Vashook V., Guth U. (2004). Response behaviour of perovskites and Au/oxide composites as HC-electrodes in different combustibles. Solid State Ionics.

[8] Azad A.M., Akbar S.A., Mhaisalkar S.G., Birkefeld L.D., Goto K.S. (1992). Solid-state gas sensors: a review. J. Electrochem. Soc..

[9] Moseley P. (1997). Solid state gas sensors. Meas. Sci. Technol..

[10] Riegel J., Neumann H., Wiedenmann H.-M. (2002). Exhaust gas sensors for automotive emission control. Solid State Ionics.

[11] Fergus J.W. (2007). Solid electrolyte based sensors for the measurement of CO and hydrocarbon gases. Sens. Actuat. B-Chem..

[12] Guth U., Zosel J. (2004). Electrochemical solid electrolyte gas sensors - hydrocarbon and NOx analysis in exhaust gases. Ionics.

[13] Park CO., Akbar S.A., Weppner W. (2003). Ceramic electrolytes and electrochemical sensors. J. Mater. Sci..

[14] Möbius H.-H., Göpel W., Hesse J., Zemel J.N. (1991). Solid-state electrochemical potentiometric sensors for gas analysis. Sensors.

[15] Möbius H., Sandow H, Hartung R, Jakobs S, Guth U., Buhrow J. (1992). Entwicklung neuer sensorsysteme mit galvanischen hochtemperatur-festelektrolytzellen. DECHEMA Monographie.

[16] Harbeck W., Guth U. (1990). Ermittlung der ausbrandgrenze von gasflammen mit hilfe gaspotentiometrischer bestimmungsmethoden. Die Industriefeuerung.

[17] Möbius H.-H., Zhuk P.P., Jakobs S., Hartung R., Guth U., Sandow H., Vecher A.A. (1991). Investigation of electrochemical oxygen sensors with solid electrolytes and oxide powder electrodes. Sov. Electrochem. Tranl..

[18] Guth U., Hartung R., Jakobs S., Sandow K.-P., Westphal D. Oxides as an electrode material for solid electrolyte gas sensors.

[19] Shuk P., Vecher A., Kharton V., Tichonova L., Wiemhöfer H.D., Göpel W., Guth U. (1993). Electrodes for oxygen sensors based on rate earth manganites or cobaltites. Sens. Actuat. B-Chem..

[20] Sandow K.-P., Jakobs S., Thiemann S., Hartung R., Guth U., Schönauer U. (1996). Dotierte lanthan-chrom-mischoxide als elektrodenmaterialien für ZrO_2_-Festelektrolyte. GDCH-Monographie..

[21] Shuk P., Bailey E., Guth U. (2008). Zirconia oxygen sensors for process application: state-of-the-art sensors. Transducers.

[22] Guth U., Bard A.J., Inzelt G., Scholz F. (2008). Gas sensors. Electrochemical Dictionary.

[23] Westphal D., Jakobs S., Guth U. (2001). Gold-composite electrodes for hydrocarbon sensors based on YSZ solid electrolytes. Ionics.

[24] Miura N., Raisen T., Lu G., Yamazoe N. (1998). Highly selective CO sensor using stabilized zirconia and a couple of oxide electrodes. Sens. Actuat. B-Chem..

[25] Hartung R., Schröder R., Möbius H.-H. (1981). Brenngas-sensitive gassymmetrische galvanische Zellen mit oxidionenleitenden Festelektrolyten. Z. Phys. Chem..

[26] Guth U., Zosel J., Jakobs S., Westphal D., Müller R. (2002). Au–oxide composites as HC-sensitive electrode material for mixed potential gas sensors. Solid State Ionics.

[27] Wang J., Elumalai P., Terada D., Hasei M., Miura N. (2006). Mixed-potential-type zirconia-based NOx sensor using Rh-loaded NiO sensing electrode operating at high temperatures. Solid State Ionics.

[28] Chevallier L., Di Bartolomeo E., Grilli ML., Mainas M., White B., Wachsman E.D., Traversa E. (2008). Non-Nernstian planar sensors based on YSZ with a Nb_2_O_5_ electrode. Sens. Actuat. B-Chem..

[29] Morata A., Viricelle J.P., Tarancon A., Dezanneau G., Pijolat C., Peiro F., Morante J.R. (2008). Development and characterisation of a screen-printed mixed potential gas sensor. Sens. Actuat. B-Chem..

[30] Miura N., Nakatou M., Zhuiykov S. (2003). Impedancemetric gas sensor based on stabilised zirconia solid electrolyte and oxide sensing electrode for detecting total NOx at high temperature. Sens. Actuat. B-Chem..

[31] Nakatou M., Miura N. (2006). Detection of propene by using new-type impedancemetric zirconia based sensor attached with oxide sensing-electrode. Sens. Actuat. B-Chem..

[32] Zosel J., Franke D., Ahlborn K., Gerlach F., Vashook V., Guth U. (2008). Perovskite related electrode materials with enhanced NO sensitivity for mixed potential sensors. Solid State Ionics.

[33] Di Bartolomeo E., Grilli ML., Traversa E. (2004). Sensing mechanism of potentiometric gas sensors based on stabilized zirconia with oxide electrodes. J. Electrochem. Soc..

[34] Zosel J., Tuchtenhagen D., Ahlborn K., Guth U. (2008). Mixed potential gas sensor with short response time. Sens. Actuat. B-Chem..

[35] Plashnitsa V.V., Ueda T., Elumalai P., Miura N. (2008). NO_2_ sensing performances of planar sensor using stabilized zirconia and thin-NiO sensing electrode. Sens. Actuat. B-Chem..

[36] Nakatou M., Miura N. (2005). Detection of combustible hydrogen-containing gases by using impedancemetric zirconia-based water-vapor sensor. Solid State Ionics.

[37] Miura N., Wang J., Nakatou M., Elumalai P., Hasei M. (2005). NO_x_ sensing characteristics of mixed-potential-type zirconia sensor using NiO sensing electrode at high temperatures. Electrochem. Solid-State Lett..

[38] Available on line at www.zirox.de

[39] Zosel J., Opitz T., Bley T., Guth U. (2006). Application of highly sensitive gas sensors for monitoring of mould culture. Der Versuchs- und Forschungsingenieur.

[40] Rettig F., Moos R. (2007). Direct thermoelectric gas sensors: design aspects and first gas sensors. Sens. Actuat. B-Chem..

[41] Rettig F., Moos R. (2009). Temperature modulated direct thermoelectric gas sensors: thermal modeling and results for fast hydrocarbon sensors. Meas. Sci. Technol..

[42] Moos R., Gnudi A., Härdtl K.H. (1995). Thermopower of Sr_1-x_La_x_TiO_3_ ceramics. J. Appl. Phys..

[43] Jonker G.H. (1968). Application of combined conductivity and seebeck-effect plots for analysis of semiconductor properties. Philips Res. Rep..

[44] Moos R. (1998). Verfahren und Meßwandler zur Detektion des Sauerstoffgehaltes in einem Gas. German Patent Specification, DE 19853595 C1.

[45] Ionescu R. (1998). Combined seebeck and resistive SnO_2_ gas sensors, a new selective device. Sens. Actuat. B-Chem..

[46] Liess M., Steffes H. (2000). The modulation of thermoelectric power by chemisorption - a new detection principle for microchip chemical sensors. J. Electrochem. Soc..

[47] Moos R., Härdtl K.H. (1995). Dependence of the intrinsic conductivity minimum of SrTiO_3_ ceramics on the sintering atmosphere. J. Amer. Ceram. Soc..

[48] Choi G.M., Tuller H.L. (1988). Defect structure and electrical properties of single-crystal Ba_0.03_Sr_0.97_TiO_3_. J. Amer. Ceram. Soc..

[49] Moos R., Menesklou W., Schreiner H.J., Härdtl K.H. (2000). Materials for temperature independent resistive oxygen sensors for combustion exhaust gas control. Sens. Actuat. B-Chem..

[50] Sahner K., Schönauer D., Matam M., Post M., Moos R. (2008). Selectivity enhancement of p-type semiconducting hydrocarbon sensors - The use of sol precipitated nano-powders. Sens. Actuat. B-Chem..

[51] Rettig F., Moos R. (2008). Morphology dependence of thermopower and resistance in semiconducting oxides with space charge regions. Solid State Ionics.

[52] Rettig F., Moos R. (2007). Direct thermoelectric hydrocarbon gas sensors based on SnO_2_. IEEE Sensors J..

[53] Ahlgren E.O., Poulsen F.W. (1995). Thermoelectric power of stabilized zirconia. Solid State Ionics.

[54] Röder-Roith U., Rettig F., Röder T., Janek J., Moos R., Sahner K. (2009). Thick-film solid electrolyte oxygen sensors using the direct ionic thermoelectric effect. Sens. Actuat. B-Chem..

[55] Stetter J.R., Li J. (2008). Amperometric gas sensors - A review. Chem. Rev..

[56] Turner A.P.F., Karube I., Wilson G.S. (1987). Biosensors: Fundamentals and Applications.

[57] Mitsubayashi K., Yokoyama K., Takeuchi T., Karube I. (1994). Gas-phase biosensor for ethanol. Anal. Chem..

[58] Mitsubayashi K., Nishio G., Sawai M., Saito T., Kudo H., Saito H., Otsuka K., Noguer T., Marty J.L. (2008). A bio-sniffer stick with FALDH (formaldehyde dehydrogenase) for convenient analysis of gaseous formaldehyde. Sens. Actuat. B-Chem..

[59] Achmann S., Hämmerle M., Moos R. (2008). Amperometric enzyme-based biosensor for direct detection of formaldehyde in the gas phase: Dependence on electrolyte composition. Electroanalysis.

[60] Hämmerle M., Achmann S., Moos R. (2008). Gas diffusion electrodes for use in an amperometric enzyme biosensor. Electroanalysis.

[61] Achmann S., Hämmerle M., Moos R. (2008). Amperometric enzyme-based gas sensor for formaldehyde: Impact of possible interferences. Sensors.

[62] Achmann S., Hämmerle M., Kita J., Moos R. (2008). Miniaturized low temperature co-fired ceramics (LTCC) biosensor for amperometric gas sensing. Sens. Actuat. B-Chem..

[63] Heiland G. (1957). Zum Einfluss von Wasserstoff auf die elektrische Leitfähigkeit an der Oberfläche von Zinkoxydkristallen. Z. Phys..

[64] Seiyama T., Kato A., Fujiishi K., Nagatani M. (1962). A new detector for gaseous components using semiconductive thin films. Anal. Chem..

[65] Taguchi N. (1956). Gas-detecting device.

[66] Eranna G., Joshi B., Runthala D., Gupta R. (2004). Oxide materials for development of integrated gas sensors - a comprehensive review. CRC Crit. Rev. Sol. St. Mat. Sci..

[67] Korotcenkov G. (2007). Metal oxides for solid-state gas sensors: What determines our choice?. Mater. Sci. Eng. B-Solid State M..

[68] Barsan N., Weimar U. (2003). Understanding the fundamental principles of metal oxide based gas sensors; the example of CO sensing with SnO_2_ sensing in the presence of humidity. J. Phys. Condens. Matter.

[69] Barsan N., Weimar U. (2001). Conduction model of metal oxide gas sensors. J. Electroceram..

[70] Barsan N., Koziej D., Weimar U. (2007). Metal oxide based gas sensor research: how to?. Sens. Actuat. B-Chem..

[71] Kappler J., Tomescu A., Barsan N., Weimar U. (2001). CO consumption of Pd doped SnO_2_ based sensors. Thin Solid Films.

[72] Emiroglu S., Barsan N., Weimar U., Hoffmann V. (2001). In situ diffuse reflectance infrared spectroscopy study of CO adsorption on SnO_2_. Thin Solid Films.

[73] Harbeck S., Szatvanyi A., Barsan N., Weimar U., Hoffmann V. (2003). DRIFT studies of thick film un-doped and Pd-doped SnO2 sensors: temperature changes effect and CO detection mechanism in the presence of water vapour. Thin Solid Films.

[74] Sahm T., Gurlo A., Barsan N., Weimar U., Madler L. (2005). Fundamental studies on SnO_2_ by means of simultaneous work function change and conduction measurements. Thin Solid Films..

[75] Koziej D., Barsan N., Weimar U., Szuber J., Shimanoe K., Yamazoe N. (2005). Water-oxygen interplay on tin dioxide surface: Implication on gas sensing. Chem. Phys. Lett..

[76] Sahm T., Mädler L., Gurlo A., Barsan N., Pratsinis S.E., Weimar U. (2004). Flame spray synthesis of tin dioxide nanoparticles for gas sensing. Sens. Actuat. B-Chem..

[77] Schmid W., Barsan N., Weimar U. (2004). Sensing of hydrocarbons and CO in low oxygen conditions with tin dioxide sensors: possible conversion paths. Sens. Actuat. B-Chem..

[78] Schmid W., Barsan N., Weimar U. (2003). Sensing of hydrocarbons with tin oxide sensors: possible reaction path as revealed by consumption measurements. Sens. Actuat. B-Chem..

[79] Bertrand J., Koziej D., Barsan N., Viricelle J.P., Pijolat C., Weimar U. (2006). Influence of the nature of the electrode on the sensing performance of SnO_2_ sensors; Impedance spectroscopy studies. Eurosensors XX.

[80] Bertrand J., Viricelle J.P., Pijolat C., Haensch A., Koziej D., Barsan N., Weimar U. Metal/SnO_2_ interface effects on CO sensing; operando studies. IEEE Sensors.

[81] Gurlo A., Barsan N., Oprea A., Sahm M., Sahm T., Weimar U. (2004). An n- to p-type conductivity transition induced by oxygen adsorption on alpha-Fe_2_O_3_. Appl. Phys. Lett..

[82] Pokhrel S., Simion C.E., Quemener V., Barsan N., Weimar U. (2008). Investigations of conduction mechanism in Cr_2_O_3_ gas sensing thick films by ac impedance spectroscopy and work function changes measurements. Sens. Actuat. B-Chem..

[83] Hernandez-Ramirez F., Prades J.D., Tarancon A., Barth S., Casals O., Jiménez-Diaz R., Pellicer E., Rodriguez J., Juli M.A., Romano-Rodriguez A., Morante J.R., Mathur S., Helwig A., Spannhake J., Mueller G. (2007). Portable microsensors based on individual SnO_2_ nanowires. Nanotechnology.

[84] Hernandez-Ramirez F., Prades J.D., Tarancon A., Barth S., Casals O., Jiménez-Diaz R., Pellicer E., Rodriguez J., Morante J.R., Juli M.A., Mathur S., Romano-Rodriguez A. (2008). Insight into the role of oxygen diffusion in the sensing mechanisms of SnO_2_ nanowires. Adv. Funct. Mater..

[85] Polleux J., Gurlo A., Barsan N., Weimar U., Antonietti M., Niederberger M. (2006). Template-free synthesis and assembly of single-crystalline tungsten oxide nanowires and their gas-sensing properties. Angew. Chem. Int. Ed..

[86] Yannopoulos L.N. (1987). A p-type semiconducting thick film gas sensor. Sens. Actuat..

[87] Carotta M.C., Martinelli G., Sadaoka Y., Nunziante P., Traversa E. (1998). Gas-sensitive electrical properties of perovskite-type SmFeO_3_ thick films. Sens. Actuat. B-Chem..

[88] Morrison S. R. Selectivity in semiconductor sensors.

[89] Moseley P., Williams D.E. (1989). Gas sensors based on oxides of early transition metals. Polyhedron..

[90] Niemeyer D., Williams D.E., Smith P., Pratt K.F., Slater B., Catlow C.R.A., Stoneham A.M. (2002). Experimental and computational study of the gas-sensor behaviour and surface chemistry of the solid-solution Cr_2-x_Ti_x_O_3_ (x < 0.5). J. Mater. Chem..

[91] Aono H., Traversa E., Sakamoto M., Sadaoka Y. (2003). Crystallographic characterization and NO_2_ gas sensing property of LnFeO_3_ prepared by thermal decomposition of Ln-Fe hexacyanocomplexes, Ln[Fe(CN)_6_]*n*H_2_O, Ln = La, Nd, Sm, Gd, and Dy. Sens. Actuat. B-Chem..

[92] Moos R., Rettig F., Hürland A., Plog C. (2003). Temperature-independent resistive oxygen exhaust gas sensors for lean-burn engines in thick-film technology. Sens. Actuat. B-Chem..

[93] Rothschild A., Litzelmann S.J., Tuller H.L., Menesklou W., Schneider T., Ivers-Tiffée E. (2005). Temperature-independent resistive oxygen sensors based on SrTi_1-x_Fe_x_O_3-δ_ solid solutions. Sens. Actuat. B-Chem..

[94] Rettig F., Moos R., Plog C. (2003). Sulfur adsorber for thick-film exhaust gas sensors. Sens. Actuat. B-Chem..

[95] Rettig F., Moos R., Plog C. (2004). Poisoning of temperature independent resistive oxygen sensors by sulfur dioxide. J. Electroceram..

[96] Blase R., Härdtl K.H., Schönauer U. (1997). Oxygen Sensor based on non-doped cuprate.

[97] Moos R., Rettig F. (2003). Resistiver sauerstoffsensor (Resistive Oxygen Sensor).

[98] Sahner K., Moos R., Izu N., Shin W., Murayama N. (2006). Response kinetics of temperature independent resistive oxygen sensor formulations: a comparative study. Sens. Actuat. B-Chem..

[99] Sahner K., Straub J., Moos R. (2006). Cuprate-ferrate compositions for temperature independent resistive oxygen sensors. J. Electroceram..

[100] Sahner K., Moos R., Matam M., Tunney J., Post M. (2005). Hydrocarbon sensing with thick and thin film p-type conducting perovskite materials. Sens. Actuat. B-Chem..

[101] Sahner K., Schönauer D., Moos R., Matam M., Post M.L. (2006). Effect of electrodes and zeolite cover layer on hydrocarbon sensing with p-type perovskite SrTi_0.8_Fe_0.2_O_3-δ_ thick and thin films. J. Mater. Sci..

[102] Sahner K., Gouma P., Moos R. (2007). Electrodeposited and sol-gel precipitated p-type SrTi_1-x_FexO_3-δ_ semiconductors for gas sensing. Sensors.

[103] Sahner K., Moos R. (2007). Modeling of hydrocarbon sensors based on p-type semiconducting perovskites. Phys. Chem. Chem. Phys..

[104] Sahner K., Moos R. (2008). P-type semiconducting perovskite sensors for reducing gases – model description. Sens. Lett..

[105] Weitkamp J. (2000). Zeolites and catalysis. Solid State Ionics.

[106] Sahner K., Hagen G., Schönauer D., Reiß S., Moos R. (2008). Zeolites - Versatile materials for gas sensors. Solid State Ionics.

[107] Xu X., Wang J., Long Y. (2006). Zeolite-based materials for gas sensors. Sensors.

[108] Moos R., Sahner K., Hagen G., Dubbe A. (2006). Zeolites for sensors for reducing gases. Rare Metal Mat. Eng..

[109] Vilaseca M., Coronas J., Cirera A., Cornet A., Morante J., Santamaría J. (2003). Use of zeolite films to improve the selectivity of reactive gas sensors. Catal Today.

[110] Hugon O., Sauvan M., Benech P., Pijolat C., Lefebvre F. (2000). Gas separation with a zeolite filter, application to the selectivity enhancement of chemical sensors. Sens. Actuat. B-Chem..

[111] Sahner K., Schönauer D., Kuchinke P., Moos R. (2008). Zeolite cover layer for selectivity enhancement of p-type semiconducting hydrocarbon sensors. Sens. Actuat. B-Chem..

[112] Meier B., Werner T., Klimant I., Wolfbeis O.S. (1995). Novel oxygen sensor material based on a ruthenium bipyridil complex encapsulated in zeolite Y - dramatic differences in the efficiency of luminescence quenching by oxygen on going from surface-adsorbed to zeolite-encapsulated flurophores. Sens. Actuat. B-Chem..

[113] Nischwitz P., Amels P., Fetting F. (1994). Studies on the ionic conductivity of zeolitic solids. Solid State Ionics.

[114] Plog C., Maunz W., Kurzweil P., Obermeier E., Scheibe C. (1995). Combustion gas sensitivity of zeolite layers on thin-film capacitors. Sens. Actuat. B-Chem..

[115] Schäf O., Ghobarkar H., Guth U. (1997). Sensors for combustible gas components using modified single crystal zeolites. Ionics.

[116] Schäf O., Ghobarkar H., Steinbach A.C., Guth U. (2000). Basic investigations on zeolite application for electrochemical analysis. Fresenius J. Anal. Chem..

[117] Neumeier S., Echterhof T., Bölling R., Pfeifer H., Simon U. (2008). Zeolite based trace humidity sensor for high temperature applications in hydrogen atmosphere. Sens. Actuat. B-Chem..

[118] Simon U., Flesch U., Maunz W., Müller R., Plog C. (1998). The effect of NH_3_ on the ionic conductivity of dehydrated zeolites Na beta and H beta. Micropor. Mesopor. Mat..

[119] Franke M., Simon U., Moos R., Knezevic A., Müller R., Plog C. (2003). Development and working principle of an ammonia gas sensor based on a refined model for solvate supported proton transport in zeolites. Phys. Chem. Chem. Phys..

[120] Moos R., Müller R., Plog C., Knezevic A., Leye H., Irion E., Braun T., Marquardt K., Binder K. (2002). Selective ammonia exhaust gas sensor for automotive applications. Sens. Actuat. B-Chem..

[121] Rodriguez-Gonzalez L., Franke M., Simon U. (2005). Electrical detection of different amines with proton-conductive H-ZSM-5. Molecular Sieves: from basic research to industrial applications.

[122] Schönauer D., Sichert I., Moos R. (2008). Zeolithe zur Ammoniakdetektion in Abgasen. Z. Anorg. Allg. Chem..

[123] Tennison P., Lambert C., Levin M. (2004). NO_x_ control development with urea SCR on a diesel passenger car.

[124] Hagen G., Dubbe A., Rettig F., Jerger A., Birkhofer T., Müller R., Plog C., Moos R. (2006). Selective impedance based gas sensors for hydrocarbons using ZSM-5 zeolite films with chromium(III)oxide interface. Sens. Actuat. B-Chem..

[125] Hagen G., Schulz A., Knörr M., Moos R. (2007). Four-wire impedance spectroscopy on planar zeolite/chromium oxide based hydrocarbon gas sensors. Sensors.

[126] Reiß S., Hagen G., Moos R. (2008). Zeolite-based Impedimetric Gas Sensor Device in Low-cost Technology for Hydrocarbon Gas Detection. Sensors.

[127] Fischerauer A., Gollwitzer A., Thalmayr F., Hagen G., Moos R., Fischerauer G. (2008). An initial physics-based model for the impedance spectrum of a hydrocarbon sensor with a zeolite/Cr_2_O_3_ interface. Sens. Lett..

[128] Achmann S., Hagen G., Kita J., Malkowsky I.M., Kiener C., Moos R. (2009). Metal-organic frameworks for sensing applications in the gas phase. Sensors.

[129] Sauerwald T., Skiera D., Kohl C.-D. (2007). Selectivity enhancement of gas sensors using non-equilibrium polarisation effects in metal oxide films. Appl. Phys. A.

[130] Waitz T., Wagner T., Sauerwald T., Kohl C.-D., Tiemann M. (2009). Ordered mesoporous In_2_O_3_: synthesis by structure replication and application as a methane gas sensor. Adv. Funct. Mater..

[131] Wagner T., Sauerwald T., Kohl C.-D., Waitz T., Weidmann C., Tiemann M. (2009). Gas sensor based on ordered mesoporous In_2_O_3_. Thin Solid Films.

[132] Wessels J.M., Nothofer H.-G., Ford W.E., von Wrochem F., Scholz F., Vossmeyer T., Schroedter A., Weller H., Yasuda A. (2004). Optical and electrical properties of three-dimensional interlinked gold nanoparticle assemblies. J. Amer. Chem. Soc..

[133] Joseph Y., Guse B., Yasuda A., Vossmeyer T. (2005). Chemiresistor coatings from Pt- and Au-nanoparticle/nonanedithiol films: sensitivity to gases and solvent vapors. Sens. Actuat. B-Chem..

[134] Krasteva N., Fogel Y., Bauer R.E., Müllen K., Joseph Y., Matsuzawa N., Yasuda A., Vossmeyer T. (2007). Vapor sorption and electrical response of Au-nanoparticle-dendrimer composites. Adv. Funct. Mater..

[135] Sahner K., Tuller H.L. (2009). Novel deposition techniques for metal oxides: prospects for gas sensing. J. Electroceram..

[136] Mädler L., Sahm T., Gurlo A., Barsan N., Grunwald J.D., Weimar U., Pratsinis S.E. (2006). Sensing low concentrations of CO using flame-spray-made Pt/SnO_2_ nanoparticles. J. Nanoparticle Res..

[137] Sahm T., Rong W., Barsan N., Mädler L., Weimar U. (2007). Sensing of CH_4_, CO and ethanol with in situ nanoparticle aerosol-fabricated multilayer sensors. Sens. Actuat. B-Chem..

[138] Sahner K., Kaspar M., Moos R. (2009). Assessment of the aerosol deposition method for preparing metal oxide gas sensors at room temperature. Sens. Actuat. B-Chem..

[139] Akedo J., Lebedev M. (1999). Microstructure and electrical properties of lead zirconate titanate (Pb(Zr_0.52_Ti_0.48_)O_3_) thick film deposited with aerosol deposition method. Jpn. J. Appl. Phys..

[140] Koplin T.J., Siemons M., Océn-Valéntin C., Sanders D., Simon U. (2006). Workflow for high throughput screening of gas sensing materials. Sensors.

[141] Frenzer G., Frantzen A., Sanders D., Simon U., Maier W.F. (2006). Wet chemical synthesis and screening of thick porous oxide films for resistive gas sensing applications. Sensors.

[142] Siemons M., Leifert A., Simon U. (2007). Preparation and gas sensing characteristics of nanoparticulate p-type semiconducting LnFeO_3_ and LnCrO_3_ materials. Adv. Funct. Mater..

[143] Siemons M., Koplin T.J., Simon U. (2007). Advances in high throughput screening of gas sensing materials. Appl. Surf. Sci..

[144] Lundtström I., Shivaraman M.S., Svenson C.M. (1975). Hydrogen-sensitive Pd-gate MOS-transistor. J. Appl. Phys..

[145] Zhang T.H., Petelenz D., Janata J. (1993). Temperature-controlled Kelvin microprobe. Sens. Actuat. B-Chem..

[146] Lorenz H., Perschke M., Riess H., Janata J., Eisele I. (1990). New suspended gate FET technology, for physical deposition of chemically sensitive layers. Sens. Actuat. A-Phys..

[147] Flietner B., Doll T., Eisele I. (1994). Reliable hybrid GasFETs for work-function measurements with arbitrary materials. Sens. Actuat. B-Chem..

[148] Gergintschew Z., Kornetzky P., Schipanski D. (1996). The capacitively controlled field effect transistor (CCFET) as a new low power gas sensor. Sens. Actuat. B-Chem..

[149] Pohle R., Simon E., Fleischer M., Meixner H., Frerichs H.-P., Lehmann M., Verhoeven H. Realisation of a new sensor concept: improved CCFET and SGFET type gas sensors in hybrid flip-chip technology.

[150] Fleischer M., Ostrick B., Pohle R., Simon E., Meixner H., Bilger C., Daeche F. (2001). Low-power gas sensors based on work function measurement in low-cost hybrid flip-chip technology. Sens. Actuat. B-Chem..

[151] Schneider R., Fleischer M., Lampe U., Pohle R., Simon E. Integrated sensor array based on field effect transistors for the detection of different gas components.

[152] Ostrick B., Pohle R., Fleischer M., Meixner H. (2000). TiN in Workfunction Type Sensors: A stable ammonia sensitive material for room temperature operation with low humidity cross sensitivity. Sens. Actuat. B-Chem..

[153] Oprea A., Simon E., Fleischer M., Lehmann M., Frerichs H.-P., Weimar U. (2005). Flip-chip suspended gate field effect transistor for ammonia detection. Sens. Actuat. B-Chem..

[154] Eisele I., Doll T., Burgmair M. (2001). Low power gas detection with FET sensors. Sens. Actuat. B-Chem..

[155] Scharnagl K., Eriksson M., Karthigeyan A., Burgmair M., Zimmer M., Eisele I. (2001). Hydrogen detection at high concentrations with stabilised palladium. Sens. Actuat. B-Chem..

[156] Senft C., Galonska T., Widanarto W., Frerichs H-P., Wilbertz C., Eisele I. Stability of FET - based hydrogen sensors at high temperatures.

[157] Zimmer M., Burgmair M., Scharnagl K., Karthigeyan A., Doll T., Eisele I. (2001). Gold and platinum as ozone sensitive layer in work-function gas sensors. Sens. Actuat. B-Chem..

[158] Sulima T., Knittel T., Freitag G., Widanarto W., Eisele I. A gas FET for chlorine detection.

[159] Stegmeier S. (2007). Nanotechnologische Schichten zur Detektion von flüchtigen Kohlenwasserstoffen mit SGFET- Gassensoren. Bachelor Thesis.

[160] Stegmeier S., Hauptmann P., Fleischer M. Room temperature detection of VOCs with activated platinum and supported platinum sensing layers by the change of work function.

[161] Kiss G., Josepovits V.K., Kovacs K., Ostrick B., Fleischer M., Meixner H., Reti F. CO Sensitivity of the PtO/SnO2 and PdO/SnO2 layer structures. Kelvin probe and XPS analysis.

[162] Lampe U., Simon E., Pohle R., Fleischer M., Meixner H., Frerichs H.-P., Lehmann M., Kiss G. (2005). GasFET for the detection of reducing gases. Sens. Actuat. B-Chem..

[163] Ostrick B., Mühlsteff J., Fleischer M., Meixner H., Doll T., Kohl C.-D. (1999). Adsorbed water as key to room temperature gas-sensitive reactions in work function type gas sensors: the carbonate carbon dioxide system. Sens. Actuat. B-Chem..

[164] Ostrick B., Fleischer M., Meixner H., Kohl C.-D. (2000). Investigation of the reaction mechanisms, in work function type gas sensors at room temperature by studies of the cross sensitivity to oxygen and water: the carbonate-carbon dioxide system. Sens. Actuat. B-Chem..

[165] Ostrick B., Fleischer M., Meixner H. (2003). The influence of interfaces and interlayers on the gas sensitivity in work function type sensors. Sens. Actuat. B-Chem..

[166] Simon E., Lampe U., Pohle R., Fleischer M., Meixner H., Frerichs H.-P., Lehmann M., Verhoeven H. Novel carbon dioxide gas sensors based on field effect transistors.

[167] Stegmaier S., Fleischer M., Hauptmann P. Detection of VOC with activated Pt and supported Pt sensing layers by the change of work function at room temperature.

[168] Simon E, Fleischer M., Meixner H. Polyvinylpyrrolidone, a new material for humidity sensors using workfunction readout.

[169] Jones S.L., Kittelson J., Cowan J.O., Flannery E.M., Hancox R.J., Mclachlan C.R., Taylor D.R. (2001). The predictive value of exhaled nitric oxide measurements in assessing changes in asthma control. Am. J. Respir. Crit. Care Med..

[170] Simon E., Fleischer M., Meixner H. Porhin-dyes-high potential NO_2_-layers for asthma detection in breath in workfunction type gas sensors.

[171] Fleischer M., Simon E., Rumpel E., Ulmer H., Harbeck M., Wandel M., Fietzek C., Weimar U., Meixner H. (2002). Detection of volatile compounds correlated to human diseases through breath analysis with chemical sensors. Sens. Actuat. B-Chem..

[172] Weimar U., Simon E., Fleischer M., Frerichs H.-P., Wilbertz C., Lehmann M. (2006). Copper phthalocyanine suspended gate field effect transistors for NO_2_ detection. Sens. Actuat. B-Chem..

[173] Waitz T., Wagner T., Sauerwald T., Kohl C.-D., Tiemann M. (2009). Ordered mesoporous In2O3: synthesis by structure replication and application as a methane gas sensor. Adv. Funct. Mater..

[174] Iskra P., Senft C., Kulaga-Egger D., Sulima T., Eisele I. A concept for a GasFET for high temperature operation.

